# Studies of Cancer death rates at Different Ages in England and Wales in 1921 to 1950: Uterus, Breast and Lung

**DOI:** 10.1038/bjc.1953.28

**Published:** 1953-09

**Authors:** Percy Stocks


### BRITISH JOURNAL OF CANCER

VOL. VII SEPTEMBER, 1953 NO. 3

### STUDIES OF CANCER DEATH RATES AT DIFFERENT AGES IN ENGLAND AND WALES IN 1921 TO 1950: UTERUS, BREAST AND LUNG.

PERCY STOCKS.*

* Senior Research Fellow, British Empire Cancer Campaign.

*From the Cheshire and North Wales Branch of the British Empire Cancer Campaign, Westminster Chambers, St. Werburgh Street, Chester.*

Received for publication June 23, 1953.

THERE are several problems exercising the minds of those who are interested in the prevention and treatment of cancer on which some light might be thrown by careful study of the trend of death rates at different age groups during the last thirty years in a country where diagnostic facilities, accuracy of medical certification and methods of statistical presentation of the figures derived from it have remained fairly constant. Whilst it cannot be denied that medical certification of cancer of internal organs as cause of death is often difficult and subject to considerable errors, many of these errors have little effect upon the trends of death rates in time, provided that the conditions mentioned above are fulfilled and that large numbers are being dealt with. For cancer of the breast, uterus, rectum and stomach the Registrar-General's figures for England and Wales since 1921, suitably corrected for modifications in the technique of selecting the cause to be tabulated when more than one cause is entered on a certificate, may be used with some confidence to study changes in the rates at which people die. For cancer of the bronchus and lung also the rates can, and indeed must, be studied, but with important reservations as to changes in the completeness of recognition of the disease.

Between 1921 and 1939 the only change affecting statistical presentation of mortality from these varieties of cancer was the introduction of a new form of death certificate in 1927, but since the certifier was asked to continue to mention in the space provided on the new form any disease which contributed to the fatal issue, though not related to the main cause of death, and since no change was made in the precedence given to cancer, wherever it appeared on the certificate, by the General Register Office at that time, it is unlikely that any important effects upon the death rates resulted. In 1940, however, an important change was made in the method of selecting the main cause when more than one was mentioned, a disease certified as merely contributory and not in the direct line of causation being ignored if another well-defined condition was stated as the direct cause of death. During 1936–1939 very careful evaluation of the effects of this change upon death rates was made, and in the *Statistical Review* for 1940

(Registrar-General, 1943) " conversion ratios " were published for most diseases to indicate the amount of correction necessary when comparing death rates from 1940 onwards with those for years before 1940. For breast cancer in women, for example, the factor to be applied to a rate at all ages prior to 1935 was ·941, signifying that the new procedure resulted in about 6 per cent of deaths which would previously have been credited to breast cancer being classified from 1940 onwards to some other cause.

When it is desired to study the trend of mortality at particular ages it is not safe to assume that the correction to be applied to rates before 1940 is the same at every age, until that has been tested, as may be done by examining the ratios at different age groups between the deaths in 1939 based upon the old and new methods, tabulated for that purpose in Table 21 and Appendix B1 of the *Statistical Review* for that year. For certain diseases where it was important, such as diabetes, more exact correction factors based on the dual classification carried out during the four years 1936–1939 were used in the Registrar-General's analyses of past death rates at separate age-groups, and on page 135 of the Text volume of the *Review* for 1938–1939 it was shown that for cancer of all sites combined the conversion ratios for each sex were slightly above unity at ages before 45 and below unity at ages after, diminishing regularly from 1·17 at 5–14 to ·95 at 75 and over for males. For cancer of the breast in females it can be deduced from the tables in the *Review* for 1939 referred to above that the correcting factor diminishes progressively from ·997 at ages 25–29 to ·970 at 55–59 and ·810 at ages 85 and over. This is easy to understand because of the increasing frequency of the presence of breast cancer, in its primary or secondary manifestations, as age advances, coupled with increasing liability to die of some intercurrent disease which is certified as the direct cause of death.

Throughout these studies of the trend of death rates according to age, correction of the rates in years prior to 1940 has been carried out by means of factors derived jointly from the age-by-age comparison of the deaths in 1939 and the conversion ratio for all ages combined obtained from the dual tabulations of 1936–1939. They differ from the series of death rates in Table LXXXIX of the *Statistical Review* for 1946–1947 (Text) in that the age grouping is much more detailed, quinquennial groups being used wherever the data allowed, and that the time periods are all of 5 years and extend to 1950, thus facilitating study of the behaviour of " cohorts " of people born in successive five-year periods. The sites dealt within the present paper consist of the uterus and breast (females), bronchus and lung (males).

*Cancer of the uterus.*

Table I gives the mean annual death rates from cancer of the uterus in England and Wales by quinquennial age groups in six periods 1921–25 to 1946–50. The factors applied to the rates of years prior to 1940, to correct for changes in the rules for selecting the underlying cause of death, were, at successive age groups from 30–34 to 85 and over: ·996, ·994, ·990, ·985, ·978, ·971, ·965, ·960, ·954, ·940, ·927, ·916.

The graphs on logarithmic scale in the left-hand part of Fig. 1 show that there was a steady decline at every age group between 30 and 70 throughout the period. In the right-hand part the rates of dying from uterine cancer experienced by the " cohorts " of women born around 1865, 1875, 1885, 1895, 1900 and 1905 are

Table I.—*Cancer of Uterus. Mean Annual Death Rates per Million Living in England and Wales,* 1921 *to* 1950.

| Age group. | 1921–25. | 1926–30. | 1931–35. | 1936–40. | 1941–45. | 1946–50. |
|---|---|---|---|---|---|---|
| 0– | 0·5 | 0·6 | 0·3 | 0·6 | 0·8 | 0·7 |
| 5– | 0·2 | 0·2 | 0 | 0·4 | 0·3 | 0 |
| 10– | | | 0·1 | 0 | 0·1 | 0 |
| 15– | 2·2 | 1·7 | 0·6 | 0·4 | 0·9 | 0·7 |
| 20– | | | 1·7 | 3·4 | 2·5 | 2·7 |
| 25– | 37·8 | 37·8 | 11·2 | 10·2 | 11·3 | 12·8 |
| 30– | | | 44·0 | 32·7 | 32·8 | 30·7 |
| 35– | 149 | 143 | 122 | 96·7 | 70·2 | 60·1 |
| 40– | 267 | 267 | 230 | 209 | 170 | 122 |
| 45– | 432 | 389 | 371 | 336 | 315 | 214 |
| 50– | 595 | 524 | 469 | 448 | 421 | 361 |
| 55– | 671 | 596 | 552 | 522 | 531 | 470 |
| 60– | 793 | 706 | 621 | 591 | 576 | 536 |
| 65– | 847 | 739 | 715 | 668 | 645 | 589 |
| 70– | 882 | 853 | 770 | 719 | 719 | 659 |
| 75– | 843 | 874 | 746 | 727 | 738 | 703 |
| 80– | 709 | 756 | 766 | 755 | 645 | 712 |
| 85 and over | 596 | 654 | 535 | 625 | 595 | 618 |

Fig. 1.—Cancer of uterus : death rates in England and Wales, 1921–1950. (1) Left half of graph by period and age at death. (2) Right half by date of birth and age at death.

*Note* :—The digits 2, 3, 4, etc., indicating the vertical scale for the upper half of the figure, should be read as 20, 30, 40, etc.

compared ; and in Table II the mortality experience of each cohort as it reached successive ages is expressed in terms of that of the women born about 1885 taken as 100.

TABLE II.—*Death rates from Cancer of Uterus experienced by Women Born about* 1865, 1875, 1885, 1895, 1900 *and* 1905, *per cent of those experienced by Women Born about* 1885.

| Age group. | 1865. | 1875. | 1885. | 1895. | 1900. | 1905. |
|---|---|---|---|---|---|---|
| 35– | — | — | 100 | 82 | 65 | 47 |
| 40– | — | — | 100 | 78 | 64 | 46 |
| 45– | — | 116 | 100 | 85 | 65 | — |
| 50– | — | 117 | 100 | 81 | — | — |
| 55– | 126 | 104 | 100 | — | — | — |
| 60–64 | 132 | 110 | 100 | — | — | — |

The improvement has been consistent and very considerable. For example, women born about 1905 are dying of the disease in their early years at only half the rate experienced at the same ages by women born about 1885. This improvement might be due to (*a*) effects of the falling birth rate between 1880 and 1933 on the incidence of uterine cancer, (*b*) effects of better hygiene in reducing incidence of the disease, (*c*) absolute reduction of fatality by more successful eradication of uterine growths, or (*d*) longer survival before finally dying from the disease.

How much of the improvement can be attributed to the continuous decline in fertility which occurred between 1880 and 1918 and again between 1920 and 1933 ? There are three factors to be considered, namely changes in the proportions of women of successive cohorts who (1) remained single and who (2) married but had no children, and changes in the average number of confinements of the remainder who married and had children. In the ' Decennial Supplement on Occupational Mortality ' in 1930–32 (Registrar-General, 1938) it was shown that the death rates from cancer of the uterus and breast in single as compared with married and widowed women were considerably lower in the one case and higher in the other, as may be seen from Table III, where a similar relative comparison is also made for cancer of the cervix uteri and breast in 1950–51 from data extracted by the General Regitser Office.

TABLE III.—*Single Women per cent of All Women amongst Decedents from Cancer of Uterus and Breast, with Comparative Proportions in the Population of* 1931 *and amongst all Women Dying in* 1950.

| Age. | In census population 1931. | In deaths of 1930–32. Cancer of uterus. | In deaths of 1930–32. Cancer of breast. | In deaths of 1950–51. Cancer of cervix. | In deaths of 1950–51. Cancer of breast. | In deaths of 1950, all causes. |
|---|---|---|---|---|---|---|
| 25– | 33·0 | 13·8 | 24·8 | 8·4 | 16·2 | 27·3 |
| 35– | 19·4 | 8·6 | 21·3 | 7·4 | 15·2 | 20·0 |
| 45– | 16·7 | 8·4 | 21·1 | 5·7 | 19·7 | 17·8 |
| 55– | 15·6 | 10·4 | 20·3 | 6·9 | 19·9 | 15·5 |
| 65– | 15·8 | 10·9 | 20·5 | 5·6 | 19·3 | 14·9 |
| 75 and over | 14·7 | 9·8 | 17·3 | 9·5 | 17·8 | 15·6 |

The contrast between cancer of the uterus as a whole and cancer of the breast in their relations with the married state was pronounced at every age in 1930–32 ; and comparison with the census population shows that single women were not dying of uterine cancer at anything like the rates for other women. In 1950–51 it was possible for the first time to distinguish mortality from cancer of the cervix and corpus uteri as a result of the inquiries by the General Register Office concerning the large number of deaths certified as cancer of " uterus " without further definition, and the rates for cervix uteri show still lower proportions of single women and greater contrasts with cancer of the breast and other causes of death.

Amongst women who died of cancer of the cervix in 1950–51 at ages under 55 the proportions who had never married were about one-third of the corresponding proportions amongst women who died of all causes. At ages 55–74 the proportion was about two-fifths, and after 75 it was about three-fifths.

In the Registrar-General's *Statistical Review* for 1940–45 (Registrar-General, 1951) deaths of married women from various causes were analysed by age groups according to whether the deceased women had borne any children or not, and the resulting proportions stated at death registration not to have had a child are shown in Table IV for cancer of the uterus, breast and ovary, non-genital cancer and all causes. From this source of information it appears that in 1940–45 amongst married women dying of cancer of the uterus as a whole at ages under 55 the proportions who had not borne children were about four-fifths of the corresponding proportions amongst women dying of cancer of non-genital sites. At ages after 55, however, there was no consistent difference, although the proportions infertile for cancer of the breast and ovary were consistently higher than for uterus at every age.

TABLE IV.—*Proportions of Married Women Dying in England and Wales during 1940–45 who were stated at Death Registration to have borne no Children.*

| | Infertile per cent of total for whom information was given. | | | | |
|---|---|---|---|---|---|
| Age group. | All causes of death. | Cancer, non-genital. | Cancer of uterus. | Cancer of breast. | Cancer of ovary. |
| 25– | 29·6 | 26·7 | 21·0 | 22·2 | 40·2 |
| 35– | 20·6 | 20·5 | 16·6 | 19·9 | 31·3 |
| 45– | 18·0 | 18·0 | 15·5 | 21·4 | 28·9 |
| 55– | 16·1 | 16·1 | 16·8 | 20·3 | 24·7 |
| 65– | 14·5 | 14·5 | 13·4 | 18·1 | 22·4 |
| 75 and over | 15·5 | 14·6 | 17·2 | 19·1 | 21·1 |

From the data given in the Registrar-General's *Statistical Reviews* for 1938–39 (Text, Appendix 1, p. 233) and 1940–45 (Text, Civil, Table xvi, p. 41) the proportions of all women born in 1885, 1890, 1895, 1900 and 1905 who had married by the time they reached successive age groups can be deduced.

The proportion who had married by 40–44 rose from about 75 per cent for the 1885 cohort to 78 for the 1900 cohort, and if the death rate from uterine cancer at 45–54 is taken as half as great in single as in other women (as was found for 1930–32 and 1910–20), this might have caused the death rate of all women to rise by 2 per cent.

According to estimations of childlessness based upon the Family Census which was carried out in 1948 for the Royal Commission on Population (1950), the proportion having at least one child within 10 years of the first marriage decreased from about 86 per cent for women who first married in 1910 to 80

per cent for those who first married in 1930. In Table V these data have been combined so as to estimate the proportions of all women born at various dates who married and had at least one child within 10 years of marriage.

TABLE V.—*Estimated Proportions of All Women born in* 1885, 1890, 1895, 1900 *and* 1905 *who, on attaining the ages stated, had been Married and who were Fertile.*

| Age group. | Estimated percentages married and fertile in each cohort. 1885. | 1890. | 1895. | 1900. | 1905. |
|---|---|---|---|---|---|
| 25– | 48·5 | 48·1 | 48·5 | 48·2 | 47·5 |
| 30– | 60·4 | 60·3 | 60·6 | 59·8 | 59·5 |
| 35– | 63·7 | 63·8 | 63·2 | 63·2 | 62·4 |
| 40–44 | 64·2 | 63·9 | 63·7 | 64·0 | — |

It is evident that, owing to increasing marriage rates which tended to counteract diminishing parity after marriage, the differences between the proportions of all women born at various times from 1885 to 1905 who experienced at least one confinement were too small to have made any appreciable contribution towards the large decrease in mortality from uterine cancer shown in Table II, even if the effect of a single confinement on incidence of that disease is important. It is necessary to go further and examine the changes in average family size, since it may be that multiplicity of confinements is the important factor. It has been found, for example, by Lombard and Potter from records in Massachusetts Hospitals as quoted by Kennaway (1948) that marriage before the age of 20 is strongly associated with incidence of cancer of the cervix.

In the ' Survey of Cancer in London ' by the British Empire Cancer Campaign, Harnett (1952) found that the records of 787 married and widowed patients with cancer of the cervix uteri and of 202 with cancer of the corpus uteri in London hospitals in 1938 and following years showed the following distribution, expressed as percentages of the totals for whom the facts were known, according to the number of children born to them :

| Number of children. | Cancer of cervix (787 cases). | Cancer of corpus (202 cases). | All cancer of uterus. |
|---|---|---|---|
| 0 | 9·0 | 21·8 | 11·6 |
| 1 | 14·5 | 29·2 | 17·5 |
| 2 | 16·8 | 15·3 | 16·5 |
| 3 | 12·4 | 14·4 | 12·9 |
| 4 | 11·6 | 7·9 | 10·8 |
| 5 or more | 35·7 | 11·4 | 30·7 |
| Total | 100·0 | 100·0 | 100·0 |

The age distributions, also expressed as percentages of the totals, were :

| | 25–. | 35–. | 40–. | 45–. | 50–. | 55 and over. | Total. |
|---|---|---|---|---|---|---|---|
| Cervix | 2·7 | 5·5 | 10·4 | 15·8 | 17·9 | 47·7 | 100·0 |
| Corpus | 2·3 | 1·8 | 3·6 | 11·2 | 15·8 | 65·3 | 100·0 |
| Uterus, all | 2·6 | 4·7 | 8·9 | 14·8 | 17·5 | 51·5 | 100·0 |

With sufficient accuracy for the purposes of this calculation, married and widowed women of these age groups in 1938–41 can be regarded as drawn from the " cohorts " of women who first married about 1930, 1925, 1920, 1915, 1910

and 1901–09; and the family size distributions to be expected in the age groups after 45 can be deduced from the data of completed families derived from the sample family census carried out for the Royal Commission in 1948, whilst those at ages before 45 can be estimated from the one per cent sample tables of the national census of 1951.

Table VIA shows, in condensed form, the distribution of family size for women of completed fertility, standardised for age at marriage, taken from Table 12 of the Preliminary Report of the Family Census in the Papers of the Royal Commission on Population (1950); and Table VIB shows the distribution of married women at four age groups under 50 according to the numbers of children born to them as stated at the Census of Great Britain in 1951 (Registrar-General, 1953).

TABLE VIA.—*Distribution of Family Size for Women in Great Britain who Married in* 1900–09, 1910, 1915, 1920 *and* 1925 *and had Completed their Childbearing (Standardised for age at Marriage).*

| Total number of live-births. | Date of first marriage 1900–09. | 1910. | 1915. | 1920. | 1925. |
|---|---|---|---|---|---|
| 0 | 11·3 | 12·1 | 15·0 | 14·2 | 16·6 |
| 1 | 14·8 | 17·0 | 21·2 | 21·8 | 25·1 |
| 2 | 18·7 | 20·5 | 23·4 | 23·6 | 25·1 |
| 3 | 15·6 | 17·1 | 15·9 | 16·1 | 14·2 |
| 4 | 12·0 | 11·1 | 9·5 | 9·5 | 7·7 |
| 5 or more | 27·6 | 22·2 | 15·0 | 14·8 | 11·3 |
| Total | 100·0 | 100·0 | 100·0 | 100·0 | 100·0 |

TABLE VIB.—*Distribution of Married Women aged between* 25 *and* 45 *in England and Wales at the Census of* 1951 *according to Number of Children Born to them.*

| Number of children. | Age group at census. 25–34. | 35–39. | 40–44. | 45–59. |
|---|---|---|---|---|
| 0 | 21·0 | 14·3 | 17·3 | 20·6 |
| 1 | 34·6 | 27·3 | 26·6 | 25·8 |
| 2 | 28·0 | 30·8 | 27·2 | 24·1 |
| 3 | 10·8 | 15·2 | 13·9 | 13·2 |
| 4 | 3·6 | 6·6 | 6·8 | 7·0 |
| 5 or more | 2·0 | 5·8 | 8·2 | 9·3 |
| Total | 100·0 | 100·0 | 100·0 | 100·0 |

In order to calculate the parity distribution to be expected amongst the 787 married and widowed women with cancer of the cervix if parity had no effect upon incidence of that disease, the percentages at ages 25–34, 35–39 and 40–44 (viz. 2·7, 5·5, 10·4) are distributed according to the first three columns of Table VIB, and the percentages at ages 45–49, 50–54, 55 and over are distributed according to the 3rd, 2nd and 1st columns of Table VIA. Aggregating the resulting proportions for each parity group, and carrying out the same process for the 202 married and widowed women with cancer of the corpus uteri, the expected distributions and the ratios of actual to expected numbers are as shown in Table VII.

The contrast between cervix and corpus uteri in their relations with number of children born is very pronounced. It would seem that the risk of subsequent cancer of the cervix begins to increase after 3 confinements, the 4th carrying a

TABLE VII.—*Expected and Actual Parity Distribution in Patients with Uterine Cancer.*

| Number of children. | Expected parity distributions in the patients with cancer of— Cervix. | Corpus. | Ratio of actual to expected numbers. Cervix. | Corpus. | All. |
|---|---|---|---|---|---|
| 0 | 13·1 | 12·3 | 0·69 | 1·77 | 0·91 |
| 1 | 18·6 | 17·0 | 0·78 | 1·72 | 0·97 |
| 2 | 21·6 | 20·2 | 0·77 | 0·76 | 0·77 |
| 3 | 15·6 | 15·7 | 0·79 | 0·92 | 0·83 |
| 4 | 10·4 | 11·1 | 1·12 | 0·71 | 1·04 |
| 5 or more | 20·7 | 23·7 | 1·72 | 0·48 | 1·47 |
| Total | 100·0 | 100·0 | | | |

greater risk than any of those preceding and later confinements adding still more, so that, on the average, women with 5 or more children appear to have twice as great a liability to this form of cancer as women with 1, 2 or 3 children. Cancer of the corpus uteri, however, is most likely to develop in married women who have not had more than one child, and is much less likely to be found amongst women who have experienced four or more confinements.

Since cancers of the cervix comprise about four-fifths of all uterine cancers, the feature of fertility likely to be most important in its effects upon national death rates from cancer of the uterus as a whole is a change in the proportion of married women bearing 5 or more children. In Table VIA it is shown that in the course of 20 years the proportion of married women having 4 children fell from 12 to 8 per cent, and the proportion who had 5 or more fell from about 27 to 11 per cent. Applying the incidence ratios for all uterine cancer to the distributions in Table VIA, the expected effect of the change in family size during the 20 years would be as follows :

| | Date of marriage. 1900–09. | 1910. | 1915. | 1920. | 1925. |
|---|---|---|---|---|---|
| Mortality index | 104·5 | 101·7 | 97·4 | 97·2 | 95·2 |

For comparative purposes the five groups may be regarded as equivalent to women born around 1880, 1885, 1890, 1895 and 1900, and Table II shows that the relative decline in death rates at ages 45–54 actually observed to occur as between the women born in 1875 and 1895 was about (116–83)/116 or 28 per cent, and as between the women born in 1880 and 1900 it must have been about (108–65)/108 or 40 per cent. Since the expected fall was (104·5–95·2)/104·5 or 8·4 per cent, it is evident that changes in family size can only account for a fraction, tentatively estimated as a quarter, of the actual fall in death rates from uterine cancer which has occurred. The remainder of the decline must be attributed to other factors such as improved hygiene amongst women and more effective treatment.

The clinical data on which these conclusions are founded are limited, and they need to be supported by more records of parity amongst patients with uterine cancer. More data of this kind are now in process of collection by the Cheshire and North Wales Branch of the British Empire Cancer Campaign in the Liverpool Hospital Region and North Wales.

*Cancer of the breast.*

It is sometimes argued that death rates from cancer are subject to so many errors arising in diagnosis, certification of the cause of death and subsequent translation of it into official statistics that they are poor material on which to base important conclusions about the disease. The implication is that it would be better to wait until we are in a position to have every case verified by biopsy or autopsy and to have the pathologists' findings considered by advisory committees. Whether the resulting statistics would be substantially different is still open to argument, and in any case such a position is never likely to be reached.

Cancer of the breast is subject to fewer errors of diagnosis, but owing to its tendency to chronicity it is often associated with the presence of other diseases, so that if the processes of derivation of statistics from medical certificates of cause of death were as faulty as some believe, no close argreement between the death rates emanating from the national statistical offices of two independent countries could be expected. It is well, therefore, that a test should be made by comparing the published death rates at various ages in a few countries having well-developed systems of vital statistics and in a similar state of civilisation. If the rates are found to differ consistently, either the real incidence or fatality may be different or else the statistics may be a better representation of the real position in one country than another. If, however, the rates agree closely, such agreement can hardly be explained by accident and should provide grounds for greater confidence in the statistics of deaths.

The World Health Organisation (1952) published death rates according to age for breast cancer in 1949 or 1948–49 for several countries, and they are reproduced in Table VIII for Canada, England and Wales, Denmark and Holland.

TABLE VIII.—*Death Rates from Cancer of the Breast per* 100,000 *Females by Age Groups in* 1949 *or* 1948–49.

| Age group. | Canada, 1949. | England and Wales, 1949. | Denmark, 1948–49. | Holland, 1948–49. |
|---|---|---|---|---|
| Under 25 | 0·1 | 0·0 | 0·1 | 0·0 |
| 25– | 2·8 | 3·3 | 2·5 | 0·4 |
| 35– | 21·9 | 20·6 | 17·9 | 9·0 |
| 45– | 49·6 | 49·3 | 44·1 | 34·2 |
| 55– | 78·7 | 76·3 | 76·8 | 62·8 |
| 65– | 113·6 | 111·2 | 111·7 | 106·2 |
| 75 and over | 176·3 | 175·4 | 160·9 | 188·1 |

The very close correspondence between the rates in Canada and England and Wales is remarkable when all the differences between the two countries are considered ; and at ages from 55 to 75 Denmark registered rates almost identical with those in England and Wales but appreciably lower at 35–44 and after 75. It is inconceivable that this measure of agreement could have arisen accidentally if death rates are based upon so many faults and errors as to make them unreliable. In Holland the rates at every age under 65 were so much below those of the other three countries, despite similarity of the registration and statistical systems, that a lower incidence or lower fatality in that country seems to be implied.

Table IX shows the mean annual death rates of females from breast cancer in England and Wales by quinquennial age groups in six periods from 1921–25 to 1946–50 in the same form as Table I. The factors used to correct the rates of years prior to 1940 for changes in the rules for selecting the underlying cause of death were, at successive age groups from 25–29 to 85 and over: ·997, ·992, ·987, ·983, ·979, ·975, ·970, ·960, ·940, ·915, ·880, ·845, ·810.

These rates, and the graphs in the left hand part of Fig. 2, in sharp contrast with those for uterine cancer, show on the whole very little change through 25

Fig. 2.—Cancer of breast: death rates of females in England and Wales, 1921–1950. (1) Left half of graph by period and age at death. (2) Right half by date of birth and age at death.

*Note*:—The digits 2 and 3, indicating the vertical scale for the upper part of the figure, should be read as 20 and 30.

years. At ages over 65 the rates increased up to 1940 and then fell back slightly, and between ages 30 and 45 the rates have increased since 1940. This may be seen by expressing the rates in 1936–40 as percentages of those in 1926–30, and the rates in 1946–50 as percentages of those in 1936–40 (Table X).

In the right-hand part of Fig. 2 the rates of dying from breast cancer experienced by the "cohorts" of women born about 1865, 1875, 1885 and 1895 are depicted. The graphs run together in a manner very different from those for the uterus in Fig. 1. In Table XI the mortality suffered by each cohort on attaining successive ages is expressed in terms of that of women born about 1885 taken as 100. At ages 50–54 there has been a lowering of mortality in the successive cohorts, and the trend suggests that the fall may continue at that age period and at 55–59; but on the other hand, women born since 1900 have shown an enhanced mortality before reaching 45.

TABLE IX.—*Cancer of the Breast. Mean Annual Death Rates per Million Living in England and Wales,* 1921 *to* 1950.

| Age group. | 1921–25. | 1926–30. | 1931–35. | 1936–40. | 1941–45. | 1946–50. |
|---|---|---|---|---|---|---|
| 0– | 0·1 | 0·1 | 0·1 | 0·1 | 0·1 | 0 |
| 5– | 0 | 0 | 0 | 0 | 0 | 0 |
| 10– | 0 | 0 | 0 | 0·3 | 0 | 0 |
| 15– | 0·1 | 0·1 | 0·3 | 0·1 | 0·1 | 0·1 |
| 20– | 1·4 | 0·7 | 0·9 | 1·6 | 1·4 | 2·1 |
| 25– | 9·9 | 9·4 | 8·3 | 11·9 | 10·8 | 10·6 |
| 30– | 40·3 | 45·1 | 47·5 | 45·3 | 48·4 | 53·7 |
| 35– | 119 | 126 | 129 | 123 | 137 | 142 |
| 40– | 269 | 275 | 270 | 263 | 273 | 287 |
| 45– | 455 | 450 | 466 | 455 | 453 | 452 |
| 50– | 586 | 638 | 635 | 622 | 604 | 585 |
| 55– | 737 | 749 | 782 | 778 | 738 | 725 |
| 60– | 833 | 917 | 949 | 927 | 888 | 867 |
| 65– | 927 | 987 | 1001 | 1049 | 1052 | 1000 |
| 70– | 1138 | 1160 | 1212 | 1239 | 1163 | 1210 |
| 75– | 1410 | 1388 | 1491 | 1522 | 1385 | 1504 |
| 80– | 1796 | 1701 | 1822 | 1834 | 1767 | 1829 |
| 85 and over | 2059 | 2281 | 2357 | 2548 | 2107 | 2177 |

TABLE X.—*Cancer of Breast. Relative Variation in Death Rates at Different Ages.*

| | 30–. | 35–. | 40–. | 45–. | 50–. | 55–. | 60–. | 65–. | 70–. | 75–. | 80–84. |
|---|---|---|---|---|---|---|---|---|---|---|---|
| 1936–40 per cent of 1926–30 | 100 | 98 | 96 | 101 | 97 | 104 | 101 | 106 | 107 | 110 | 108 |
| 1946–50 per cent of 1936–40 | 118 | 115 | 109 | 99 | 94 | 93 | 94 | 95 | 98 | 99 | 100 |

TABLE XI.—*Death Rates from Cancer of the Breast amongst Women Born at Various Dates, Expressed in Terms of the Rates amongst Women Born about* 1885 *taken as* 100.

| Age group. | 1865. | 1875. | 1885. | 1895. | 1900. | 1905. | 1910. |
|---|---|---|---|---|---|---|---|
| 35– | — | — | 100 | 108 | 103 | 115 | 119 |
| 40– | — | — | 100 | 96 | 99 | 104 | — |
| 45– | — | 98 | 100 | 97 | 97 | — | — |
| 50– | — | 102 | 100 | — | — | — | — |
| 55– | 100 | 106 | 100 | — | — | — | — |
| 60–64 | 106 | 107 | 100 | — | — | — | — |

To what extent, if at all, could the unsatisfactory trend of mortality from breast cancer be explained by changes in the rates of marriage and childbearing ? Table III shows that in 1930–32 the proportion of single women amongst all women who died of breast cancer at ages between 45 and 65 was 20·7 per cent compared with 16·2 per cent in the census population ; and in 1950–51 it was 19·8 per cent compared with about 16·5 amongst women dying from all causes. The 1930–32 figures imply that the death rates of single women and of other women at 45–64 were in the ratio of 20·7/15·8 to 79·3/83·8 or 1·33 to 1 ; and similarly at ages 65–74 the ratio derived from Table III was 20·5/15·8 to 79·5/84·2 or 1·37 to 1. Since, as shown in the discussion of uterine cancer, the proportion of all women still unmarried at ages 40–44 declined from about 25 per cent for women born about 1885 to 22 per cent for women born about 1900, this change in proportion marrying might be expected to lower the breast cancer death rate after 45 from R(·25 + ·75/1·35) to R(·22 + ·78/1·35), where R is the rate amongst single women. The relative improvement to be expected during the last 20 years in mortality of women between 45 and 75 on account of the increasing amount of marriage is, therefore, only about 1 per cent of the rates.

Table IV shows that amongst married women dying of breast cancer at ages over 45 in 1940–45 the percentage proportions who were stated to have had no children were greater than amongst women dying of cancer of non-genital organs or amongst those dying of all causes. Between ages 45 and 65 the proportions infertile were about 20·8 per cent compared with 17 per cent, which implies a ratio between the breast cancer death rates of infertile and fertile married women at those ages of 20·8/17 to 79·2/83 or 1·28 to 1; and similarly at ages 65–74 the ratio was 18·1/14·5 to 81·9/85·5 or 1·30 to 1. Since, as was shown in connection with uterine cancer, infertility of married women during the first ten years of marriage increased from about 14 per cent in women who married about 1910 to 20 per cent in those who married about 1930, this change in the infertility rate after marriage would be expected to raise breast cancer rates after 45 from r(·14 + ·86/1·3) to r(·20 + ·80/1·3), where r is the rate amongst fertile married women. The increase in breast cancer mortality of married women to be expected at ages over 45 in the last 20 years would be, therefore, about 2 per cent of the rate.

Information as to the numbers of pregnancies experienced by women who later develop breast cancer at various ages is scanty, and the only data found suitable for the purpose of this study were in a tabulation by Smithers *et al.* (1952) of 383 married women treated at the Royal Cancer Hospital from 1945 to 1948. Their distribution according to number of children in association with age at onset is shown in condensed form in Table XII; and by the same procedure as for uterine cancer the data in Tables VIA and VIB have been used to calculate the parity distributions to be expected on the assumption that these patients were representative of all married women with a breast cancer appearing in

TABLE XII.—*Number of Children Born to* 383 *Married Women with Cancer of Breast* (*Royal Cancer Hospital series,* 1945–48) *Compared with Numbers Expected from Data of Tables VIA and VIB.*

| Number of children. | Actual numbers according to age at onset. | | | | | | | | |
|---|---|---|---|---|---|---|---|---|---|
| | 20–. | 35–. | 40–. | 45–. | 50–. | 55–. | 60–. | 65 and over. | Total. |
| 0 | 5 | 7 | 3 | 11 | 12 | 11 | 4 | 12 | 65 |
| 1 | 6 | 5 | 14 | 13 | 9 | 15 | 17 | 18 | 97 |
| 2 | 6 | 7 | 12 | 10 | 8 | 16 | 8 | 23 | 90 |
| 3 | — | 6 | 5 | 5 | 10 | 8 | 11 | 13 | 58 |
| 4 | — | — | 1 | 1 | 2 | 6 | 8 | 6 | 24 |
| 5 | — | — | 1 | — | 3 | 2 | 2 | 9 | 17 |
| 6 | — | — | 1 | — | 1 | — | 2 | 6 | 10 |
| 7 | — | — | 1 | 1 | — | — | — | 5 | 7 |
| 8 or more | — | — | — | — | 2 | 1 | 2 | 10 | 15 |
| Total | 17 | 25 | 38 | 41 | 47 | 59 | 54 | 102 | 383 |
| | Expected parity distribution of total at each age. | | | | | | | | |
| 0 | 1·9 | 3·0 | 5·7 | 6·8 | 6·7 | 8·8 | 6·5 | 11·5 | 51·0 |
| 1 | 2·5 | 4·2 | 8·1 | 10·3 | 10·2 | 12·5 | 9·2 | 15·1 | 72·2 |
| 2 | 3·2 | 5·1 | 8·9 | 10·3 | 11·1 | 13·8 | 11·1 | 19·1 | 82·5 |
| 3 | 2·7 | 4·3 | 6·0 | 5·8 | 7·6 | 9·4 | 9·2 | 15·9 | 60·9 |
| 4 | 2·0 | 2·8 | 3·6 | 3·2 | 4·5 | 5·6 | 6·0 | 12·2 | 39·9 |
| 5 or more | 4·7 | 5·6 | 5·7 | 4·6 | 6·9 | 8·9 | 12·0 | 28·2 | 76·5 |

1945–48. The age groups under 35, 35–39, 40–44 can be regarded as corresponding in parity approximately with married women of ages 25–34, 35–39, 40–44 at the Census of 1951 (Table VIB), and age groups 45–, 50–, 55–, 60–, 65 and over can be equated to women who married about 1925, 1920, 1915, 1910 and 1900–09 (Table VIA). The expected distributions obtained by applying the appropriate percentages in Tables VIA and VIB to the numbers of patients in each age group are shown in the lower part of Table XII.

Comparing the actual with the expected parity distributions, it appears that the risk of developing breast cancer after 45 must be considerably less amongst women who have had several children than amongst married women who have had no more than one child (Table XIII).

TABLE XIII.—*Breast Cancer. Actual and Expected Parity Distributions.*

| Number of children. | Ages under 45. | | | Ages 45 and over. | | | All ages ratio. |
|---|---|---|---|---|---|---|---|
| | Actual. | Expected. | Ratio. | Actual. | Expected. | Ratio. | |
| 0 | 15 | 10·6 | 1·42 | 50 | 40·4 | 1·24 | 1·27 |
| 1 | 25 | 14·8 | 1·69 | 72 | 57·4 | 1·25 | 1·20 |
| 2 | 25 | 17·2 | 1·45 | 65 | 65·3 | 1·00 | 1·09 |
| 3 | 11 | 13·0 | 0·85 | 47 | 47·9 | 0·98 | 0·95 |
| 4 | 1 | 8·4 | 0·16 (4 and 5 or more) | 23 | 31·5 | 0·73 | 0·60 |
| 5 or more | 3 | 16·0 | | 46 | 60·5 | 0·76 | 0·64 |
| Total | 80 | 80·0 | — | 303 | 303·0 | — | — |

Multiple pregnancies may have a protective effect ; at any rate the tentative conclusions to be drawn from these data is that 2 or 3 children diminish the likelihood of breast cancer after 45 by about one-fifth, and four or more children reduce it by about two-fifths. At ages under 45 the effect seems to be even greater, but the numbers on which the ratios are based are small, and there might be other reasons contributing to the low frequency of women with four or more children amongst patients seen during the period of childbearing.

It is desirable that this inverse correlation with number of children should be confirmed by other and larger data, and such information is in process of collection in the Liverpool Hospital Region and North Wales by the British Empire Cancer Campaign. Assuming it to be correct, what effect would the decline in family size since 1905 be expected to have upon the incidence of breast cancer amongst all married women ? This can be calculated by applying the ratios for women over 45 to the successive distributions in Table VIA and aggregating the products, as was done for uterine cancer. The relative expectations of incidence amongst women who first married about the five dates are then found to be :

| 1900–09. | 1910. | 1915. | 1920. | 1925. |
|---|---|---|---|---|
| 96·2 | 98·5 | 102·4 | 102·4 | 105·2 |

The expected increase amongst married women over 45 during the 20 years would be (105·2–96·2)/96·2 or 9 per cent of the incidence in the 1900–09 group ; and if fatality had remained constant their death rates should have risen by 9 per cent. This is a larger effect than was estimated from the deaths of married women divided into the two categories fertile and infertile, but the two estimates are not incompatible. From Table XI it appeared that death rates of all women over 45 have not risen but have shown a slight improvement, and if incidence of breast cancer has in fact increased by 8 per cent (after allowing for the estimated effect of more marriage), this would mean that some compensating influence has been at work tending to lower either the incidence or fatality of the disease. Since there is no known reason why incidence should have diminished amongst groups of women of given age and fertility, the most probable explanation would be that the efficacy of surgery and radiotherapy to prolong the lives of patients with breast cancer has not remained unchanged over a quarter of a century, but has so improved as to reduce fatality rates to about 90 per cent of their former levels through raising survival rates.

At ages before 45, if the ratios deduced from Table XII are applied to the five cohorts in Table VIA, the trend of relative incidence in women who married at the five dates would be :

| 1900–09. | 1910. | 1915. | 1920. | 1925. |
|---|---|---|---|---|
| 87·7 | 95·5 | 108·5 | 108·8 | 117·5 |

Although, as Table XI indicated, death rates at ages under 45 have risen considerably, that rise would be more than accounted for by an increase in incidence such as the figures 87·7 to 117·5 suggest, again leading to the conclusion that improved results of treatment may have prevented the death rates from rising more steeply than they have done. It must be stressed, however, that the conclusions reached above from the data of the Royal Cancer Hospital need to be confirmed when more extensive information of the same kind is available.

*Cancer of lung and bronchus.*

The increase in death rates from bronchogenic carcinoma, now known to be occurring in several countries, is a matter for concern, and it is possible that some clue to the reasons for the continued rise might be found by a careful examination of the proportions of males dying of this and of all other forms of cancer amongst those born at different years. Possible explanations which have been suggested for the continued increase are : (1) more complete recognition of the disease leading to its more frequent certification as cause of death, (2) introduction of some carcinogenic substance in the process of tobacco cultivation or manufacture, (3) increase of some carcinogenic substance in the atmosphere, (4) delayed effects of virus A influenza, (5) prevention by modern therapy of early death from pneumonia at a stage before cancer could be recognised.

There is no doubt that the first and last of these have accounted for part of the increase, but the belief that one or both of them provided the whole explanation has had to be abandoned in Denmark at least, and Clemmesen, Neilsen and Jensen (1953) now postulate the appearance of a carcinogen in the environment of Copenhagen about the period 1900–10 and in the provinces some ten years later, beginning to affect people about age 15 and taking some 20 years to develop.

The purpose of this study is to discover whether any sudden increase in mortality occurred at a particular period of time or affected particular cohorts of men or affected men of particular ages. Cancer death rates in general have been influenced by a number of disturbing factors, but since only those peculiar to lung cancer are of interest here, the method used has been to examine the behaviour of the ratio of lung cancer deaths to the total deaths from cancer of all other sites in cohorts of males, born at different periods since 1855, as they reached successive years between 1921 and 1950. Lung cancer death rates and also those from other forms of cancer tend to increase steadily with advancing age, so movements of the ratio will give no indication of that part of the upward progression which lung cancer shares with other cancer. They are superimposed upon it, and give a more sensitive indication of any special factors arising which affected the incidence of lung cancer alone as the cohorts were exposed to them through particular periods of time and then passed on to higher ages. It is necessary, however, to keep in mind what was happening to the base level of mortality on which the ratios are superimposed, and the progressive upward trend with age of death rates per 100,000 from all cancer other than of lung and bronchus is shown for different cohorts (Table XIV).

TABLE XIV.—*Death Rates from all Cancer other than of Lung and Bronchus.*

| Ages reached. | Period of birth of cohort. 1871. | 1876. | 1881. | 1886. | 1891. | 1896. | 1901. | Average per cent increase in 5 years of age. |
|---|---|---|---|---|---|---|---|---|
| 35– | — | — | — | 26 | 25 | 27 | 25 | — |
| 40– | — | — | 50 | 53 | 48 | 49 | 49 | 93 |
| 45– | — | 111 | 104 | 98 | 86 | 90 | 88 | 87 |
| 50– | 213 | 204 | 186 | 172 | 161 | 152 | — | 79 |
| 55– | 353 | 333 | 305 | 282 | 265 | — | — | 64 |
| 60– | 558 | 527 | 472 | 435 | — | — | — | 56 |
| 65– | 812 | 772 | 685 | — | — | — | — | 46 |
| 70– | 1062 | 1039 | — | — | — | — | — | 33 |
| 75–79 | 1537 | — | — | — | — | — | — | 28 |

Table XV shows the lung cancer ratios in separate years 1921 to 1950 for cohorts of men who formed the age group 15–19 in the year stated in the second line of the column in question. The years of birth of the cohorts attaining these ages in 1901 would be 1882–1886, centred at 1884 as the table indicates; in 1931 the men of this cohort would be aged 45–49, in 1933 their ages would be 47–51, and so on. In calculating the ratio for the 1884 cohort in 1933, three times the deaths at 45–49 were added to twice the deaths at 50–54 in 1933, both for lung cancer and all other cancer, and the first total divided by the second. The approximation involved in this would affect numerator and denominator similarly and would have no appreciable effect on the ratio. In the lower part of the table the ratios thus obtained have been averaged for three consecutive years, centred at every third year from 1922 to 1949.

From 1922 to 1925 the lung cancer index changed appreciably only in the cohorts born about 1864, 1869 and 1884, but in each successive triennium in the years between 1925 and 1937 it increased in every cohort. Between 1937 and 1946 the increases again became selective, affecting the later cohorts. This can be seen in Table XVI, where the triennial increases are expressed as percentages of the index at the start of the period, "*nil*" denoting no increase or a slight fall. Ages between 45 and 55 are located approximately between the broken lines; figures to the left of these relate to what was happening amongst men over 55, and figures to the right relate to ages under 45.

Between 1925 and 1928 the ratio rose sharply in the 1879 and 1884 cohorts, whose ages were then about 45, and they increased also by 10 to 20 per cent at older ages. Between 1928 and 1931 the increase was intensified in every cohort,

TABLE XV.—*Ratios of Lung Cancer to All Other Cancer in Each Year* 1921 *to* 1950 *amongst Deaths occurring in Cohorts of Males, England and Wales.*

| Central birth year | 1854. | 1859. | 1864. | 1869. | 1874. | 1879. | 1884. | 1889. | 1894. | 1899. | 1904. | 1909. |
|---|---|---|---|---|---|---|---|---|---|---|---|---|
| Aged 15–19 in . | 1871. | 1876. | 1881. | 1886. | 1891. | 1896. | 1901. | 1906. | 1911. | 1916. | 1921. | 1926. |
| *Year of death.* | | | | | | | | | | | | |
| 1921 . | ·012 | ·015 | ·019 | ·028 | ·038 | ·056 | ·054 | — | — | — | — | — |
| 1922 . | ·014 | ·015 | ·019 | ·030 | ·034 | ·038 | ·057 | — | — | — | — | — |
| 1923 . | ·012 | ·016 | ·018 | ·026 | ·035 | ·046 | ·049 | — | — | — | — | — |
| 1924 . | ·010 | ·016 | ·023 | ·031 | ·037 | ·043 | ·069 | — | — | — | — | — |
| 1925 . | ·015 | ·016 | ·020 | ·027 | ·029 | ·047 | ·050 | — | — | — | — | — |
| 1926 . | ·011 | ·014 | ·023 | ·033 | ·045 | ·048 | ·049 | ·094 | — | — | — | — |
| 1927 . | ·013 | ·017 | ·025 | ·031 | ·040 | ·058 | ·080 | ·073 | — | — | — | — |
| 1928 . | ·012 | ·018 | ·030 | ·036 | ·040 | ·065 | ·093 | ·091 | — | — | — | — |
| 1929 . | ·012 | ·017 | ·024 | ·033 | ·051 | ·067 | ·090 | ·120 | — | — | — | — |
| 1930 . | ·013 | ·027 | ·026 | ·043 | ·064 | ·077 | ·100 | ·129 | — | — | — | |
| 1931 . | — | ·022 | ·032 | ·046 | ·077 | ·097 | ·138 | ·155 | ·155 | — | | — |
| 1932 . | — | ·026 | ·035 | ·052 | ·078 | ·104 | ·142 | ·183 | ·186 | — | — | — |
| 1933 . | — | ·026 | ·034 | ·052 | ·082 | ·119 | ·175 | ·204 | ·203 | — | — | — |
| 1934 . | — | ·030 | ·038 | ·047 | ·092 | ·131 | ·175 | ·210 | ·207 | — | — | — |
| 1935 . | — | ·026 | ·041 | ·059 | ·092 | ·146 | ·188 | ·232 | ·207 | — | — | — |
| 1936 . | — | — | ·040 | ·065 | ·098 | ·148 | ·194 | ·259 | ·217 | ·228 | — | — |
| 1937 . | — | — | ·038 | ·065 | ·099 | ·151 | ·221 | ·264 | ·258 | ·247 | — | — |
| 1938 . | — | — | ·044 | ·067 | ·104 | ·167 | ·238 | ·298 | ·327 | ·277 | — | — |
| 1939 . | — | — | ·041 | ·067 | ·105 | ·152 | ·232 | ·287 | ·341 | ·356 | — | — |
| 1940 . | — | — | ·041 | ·063 | ·098 | ·163 | ·234 | ·286 | ·331 | ·340 | — | — |
| 1941 . | — | — | ·034 | ·051 | ·095 | ·171 | ·252 | ·328 | ·330 | ·331 | ·280 | — |
| 1942 . | — | — | ·042 | ·058 | ·098 | ·173 | ·263 | ·316 | ·369 | ·385 | ·344 | — |
| 1943 . | — | — | ·047 | ·070 | ·108 | ·178 | ·263 | ·337 | ·398 | ·394 | ·330 | — |
| 1944 . | — | — | ·043 | ·064 | ·104 | ·173 | ·273 | ·375 | ·449 | ·459 | ·391 | — |
| 1945 . | — | — | ·043 | ·060 | ·099 | ·175 | ·271 | ·381 | ·425 | ·501 | ·419 | — |
| 1946 . | — | — | ·044 | ·061 | ·092 | ·192 | ·296 | ·427 | ·502 | ·542 | ·469 | ·379 |
| 1947 . | — | — | ·039 | ·068 | ·103 | ·198 | ·315 | ·422 | ·546 | ·610 | ·534 | ·437 |
| 1948 . | — | — | ·048 | ·074 | ·116 | ·205 | ·329 | ·459 | ·587 | ·637 | ·566 | ·464 |
| 1949 . | — | — | ·054 | ·077 | ·124 | ·213 | ·350 | ·486 | ·598 | ·670 | ·636 | ·480 |
| 1950 . | — | — | ·048 | ·079 | ·119 | ·210 | ·357 | ·527 | ·623 | ·740 | ·650 | ·550 |
| *Central year.* | | | | | | | | | | | | |
| 1922 . | ·013 | ·015 | ·019 | ·028 | ·036 | ·047 | ·053 | — | — | — | — | — |
| 1925 . | ·012 | ·015 | ·022 | ·030 | ·037 | ·046 | ·056 | — | — | — | — | — |
| 1928 . | ·012 | ·017 | ·026 | ·033 | ·044 | ·063 | ·088 | ·095 | — | — | — | — |
| 1931 . | — | ·025 | ·031 | ·047 | ·073 | ·093 | ·127 | ·156 | — | — | — | — |
| 1934 . | — | ·027 | ·038 | ·053 | ·089 | ·132 | ·179 | ·215 | ·206 | — | — | — |
| 1937 . | — | — | ·041 | ·066 | ·100 | ·155 | ·218 | ·274 | ·267 | ·251 | — | — |
| 1940 . | — | — | ·039 | ·060 | ·099 | ·162 | ·239 | ·300 | ·334 | ·342 | — | — |
| 1943 . | — | — | ·044 | ·064 | ·103 | ·175 | ·266 | ·343 | ·405 | ·413 | ·355 | — |
| 1946 . | — | — | ·042 | ·063 | ·098 | ·188 | ·294 | ·410 | ·491 | ·551 | ·474 | — |
| 1949 . | — | — | ·050 | ·077 | ·120 | ·209 | ·345 | ·491 | ·603 | ·682 | ·617 | — |

TABLE XVI.—*Increases in Lung Cancer Ratios in Triennial Periods (per cent of the ratio at start of the period) Occurring amongst Males of Different Birth Dates.*

| Period when rise occurred. | Central birth year of cohort. | | | | | | | | | |
|---|---|---|---|---|---|---|---|---|---|---|
| | 1859. | 1864. | 1869. | 1874. | 1879. | 1884. | 1889. | 1894. | 1899. | 1904. |
| 1922 to 1925 . | *nil* | 18 | 8 | 4 | *nil* | 5 | — | — | — | — |
| 1925 to 1928 . | 13 | 21 | 10 | 18 | 38 | 57 | — | — | — | — |
| 1928 to 1931 . | 44 | 18 | 41 | 67 | 46 | 44 | 64 | — | — | — |
| 1931 to 1934 . | 9 | 22 | 12 | 21 | 43 | 42 | 38 | — | — | — |
| 1934 to 1937 . | — | 8 | 25 | 13 | 18 | 21 | 27 | 30 | — | — |
| 1937 to 1940 . | — | *nil* | *nil* | *nil* | 4 | 10 | 10 | 25 | 33 | — |
| 1940 to 1943 . | — | 14 | 6 | 4 | 8 | 11 | 14 | 21 | 20 | — |
| 1943 to 1946 . | — | *nil* | *nil* | *nil* | 8 | 10 | 20 | 21 | 34 | 34 |
| 1946 to 1949 . | — | 19 | 22 | 22 | 11 | 17 | 20 | 23 | 24 | 30 |

exceeding 40 per cent except in that of 1864. In the next triennium the increases were similar to those between 1925 and 1928, and from 1934 to 1937 they again diminished, becoming small or absent in the next period, except in men around 45.

This pronounced wave of increase in lung cancer compared with other cancer, commencing amongst younger men before 1928 and then spreading to every age, reaching a maximum about 1931 and then diminishing by 1937 except in the younger groups, is suggestive of a stimulus to diagnosis produced by some new definition of a disease hitherto imperfectly recognised. Such an event did occur in 1926 when Barnard described bronchogenic carcinoma in a paper which undoubtedly had a great influence on recognition of the disease during the ensuing decade. It seems very probable that the raising of lung cancer death rates to higher levels at all ages during that period was largely due to more complete recognition of its presence as a result of this and subsequent pathological research.

An alternative explanation of the initial wave of increase might be that the influenza epidemic of 1918–19 initiated the carcinogenic process in a few of the millions of people who were affected, and that the disease took some 10 to 20 years to develop and reach a fatal termination. That is not a new suggestion, but no evidence for any causal connection with the epidemic has been advanced. The great epidemic was followed by smaller ones in 1922, 1924, 1927, 1929 and 1933, but none of them exceeded in magnitude one seventh of the 1918–19 outbreak, in terms of mortality ; and if Virus A had been responsible for the initial lung cancer wave, that wave would have subsided much further than it did. In fact the incidence of deaths never reached any peak ; what happened was that the rate of increase reached a temporary maximum about 1931 and then diminished. The "*nil*" increases in Table XVI from 1937 to 1940 mean that in the cohorts born before 1875 the lung cancer death rate was then rising with advancing age after 65 at about the same gradient as the death rate for all other cancer, and the actual gradient at that age and time period has been shown to be about 33 per cent increase in 5 years or 20 per cent in a triennium. The preceding explanation for what occurred between 1925 and 1937 is far more probable, but the hypothesis of influenza as a contributory factor cannot be excluded as a possibility.

From 1937 onwards sulphonamides began to be used in increasing amount, and pneumonia deaths soon began to fall. Could this have affected lung cancer rates appreciably by keeping alive longer many persons who without sulphonamides would have died of a complicating pneumonia before the cancer could be recognised ? Pneumonia death rates of males fell between 1937 and 1942 from 477 to 255 per million at ages 35–44, from 950 to 457 at 45–54 and from 1535 to 1024 at 55–64, and the fall continued till 1946. During that period the lung cancer ratio continued to rise steadily in men born about 1880, the gradient being steeper amongst men at ages around 40 and 50 than around 60, and only slight after 65. The following totals of deaths in England and Wales set definite limits, however, to the possible effect of sulphonamides, and of penicillin which came

| Certified cause. | Ages. | Number of deaths of males in— 1939. | 1940. | 1949. | 1950. | Decrease. | Increase. |
|---|---|---|---|---|---|---|---|
| Pneumonia | 34–68 | 5544 | — | 3614 | — | 1930 | — |
| Cancer of lung and bronchus | 35–69 | — | 3415 | — | 8357 | — | 4942 |

into use later, in saving men with lung cancer from dying of " pneumonia " and enabling them to die a year or so later of a recognised carcinoma.

Even if it were supposed that every man between 34 and 69 who was prevented by the new therapy from dying of pneumonia in 1949 died subsequently of lung cancer in 1950, which would be a fantastic overstatement, this would still leave an increase in the lung cancer deaths at those ages unaccounted for. If half of those saved in 1949 were really cases of lung cancer it would leave four-fifths of the increase in deaths between 1940 and 1950 unexplained. Although there is no doubt that this factor contributed something to the increase in lung cancer deaths after 1937, that contribution cannot have gone far towards explaining the total increase.

The use of the bronchoscope has also been increasing in frequency, and mass-radiography has brought to light some cases since 1946 which might have escaped recognition, but the latter would affect men of working ages mainly, and Table XV shows no evidence of such a selective effect appearing between 1946 and 1949. All these factors combined can hardly account for the continued increase since 1937 in the ratio of lung cancer to all other cancer by 7 or 8 per cent of the ratio annually in men born since 1890, and by 3 to 6 per cent annually in men born between 1875 and 1890.

Diagnostic difficulties are still great in many instances, and Smithers (1953) has shown in a careful survey that at the present time not only are large numbers of cases missed in death certification, but many cases are being incorrectly certified as lung cancer. Despite all this, he concludes, in conformity with the view now accepted by Clemmesen, Neilsen and Jensen (1953) in Denmark, that changes in accuracy of certification account for only a part of the apparent rise in incidence of the disease.

Neither the well-established connection between cigarette smoking and bronchogenic cancer incidence in hospital patients nor the statistical indications that smoke from domestic chimneys may also be concerned depend on any correspondence in upward or downward trends in time. Both factors have been part of our environment for a long time, but the chemical constituents of cigarettes and of domestic smoke may have been modified at any time, and changes in the total volumes are less important than the appearance of, or increase of, some carcinogenic substance in them. The introduction of petrol and oil-driven vehicles with rubber tyres into the environment early in the century has also to be kept in mind, although so far no firm evidence of a connection has been found. These three factors, acting separately or in combination, still seem to provide the most likely hypothesis as a basis for research into causation.

### CONCLUSION.

1. Examination of the death rates at quinquennial age groups from cancer of the uterus, experienced between 1921 and 1950 by women in England and Wales, shows that a progressive fall in mortality has occurred ; those born about 1905 died at only half the rate during the childbearing period compared with those born about 1885.

2. Amongst women who died from cancer of the cervix uteri in 1950–51 at ages under 55 the proportion who had never married was about one-third of the corresponding proportion amongst women dying from all causes ; at ages 55–74

the proportion was about two-fifths, and after 75 it was about three-fifths. Because of the increase which has occurred in the proportion marrying before 45, the death rates from uterine cancer at ages after 45 might have been expected to rise by 2 per cent in the last 20 years.

3. The distribution according to number of children born for married women over 45 with cancer of the cervix uteri differs greatly from that for married women over 45 with cancer of the corpus uteri, and also from the parity distribution to be expected on the basis of the general population of married women. It appears that the risk of subsequent cancer of the cervix begins to increase after 3 confinements, and that for women with 5 or more children it is twice as great as for women with few children. The progressive fall in family size during 20 years would be expected to have diminished the death rates by about 8 per cent of their former values, and the actual improvement recorded has been much greater than can be accounted for by fertility changes.

4. Examination of death rates from breast cancer at different ages in several countries reveals close agreement over a range of ages between Canada, England and Wales and Denmark ; but rates in Holland are lower. Study of the death rates experienced from 1921 to 1950 by successive cohorts of women in England and Wales shows a tendency for rates to fall slightly at ages between 50 and 60, but a pronounced increase in mortality before reaching 45 has occurred.

5. Death rates from breast cancer after 45 are greater amongst women who have never married, and the increase in the proportion of women marrying before 45 might be expected to have lowered the rates after 45 by 1 per cent in the last 20 years.

6. The distribution according to number of children born for hospital patients with breast cancer, in such data as are available, differs from what would be expected, and it is estimated tentatively that the bearing of 2 or 3 children reduces the likelihood of this form of cancer developing after 45 by about one-fifth and that 4 or more children reduces it by about two-fifths. From this it follows that the progressive fall in family size should have increased the incidence of breast cancer after 45 by about 9 per cent, and since the death rates have not increased at those ages, this suggests that a corresponding improvement in survival rates has been effected.

7. Examination of the changes between 1921 and 1950 in the ratio of deaths attributed to cancer of the lung and bronchus to deaths from all other cancer at the same ages in successive cohorts of males indicates that a wave of increase in the ratio occurred between 1927 and 1937, raising death rates to higher levels at every age. The most probable explanation for this is an improving recognition of bronchogenic cancer starting from the pathological researches published in 1926.

8. From 1937 to 1946 the lung cancer ratio continued to rise by about 7 per cent of the ratio annually in men born since 1890, more slowly in men born between 1875 and 1890, and inappreciably in cohorts born before that ; but after 1946 the increase again spread to all ages. Sulphonamides, and later penicillin, must have contributed something to this by preventing some men from dying from penumonia without cancer having been diagnosed and allowing them to die a year or so later of recognised lung cancer. The pneumonia statistics show, however, that this factor could not account for more than about a fifth of the actual increase recorded for lung cancer between 1940 and 1950.

9. It is concluded that much of the increase in lung cancer rates in England and Wales since 1937 must have been due to rising incidence of the disease, and possible causes of this are discussed.

### REFERENCES.

BARNARD, W. G.—(1926) *J. Path. Bact.*, **29**, 241.
CLEMMESEN, J., NEILSEN, A., AND JENSEN, E.—(1953) *Brit. J. Cancer*, **1**, 1.
HARNETT, W. L.—(1952) 'Survey of Cancer in London.' London (British Empire Cancer Campaign).
KENNAWAY, E. L.—(1948) *Brit. J. Cancer*, **2**, 177.
REGISTRAR-GENERAL—(1938) 'Decennial Supplement, 1931 : Occupational Mortality.' London (H.M. Stationery Office).—(1943) *Statistical Review for* 1940. Part 1 ; Medical. Appendix, B1. London (H.M. Stationery Office).—(1951) *Statistical Review for* 1940–45. Text, vol. 2, Civil. London (H.M. Stationery Office).—(1953) 'One per cent Sample Tables,' Part 2, Table X3. London (H.M. Stationery Office).
Royal Commission on Population—(1950) 'Reports and Selected Paper,' vol. 2, p. 99. London (H.M. Stationery Office).
SMITHERS, D. W.—(1953) *Brit. med. J.*, **1**, 1235.
*Idem*, RIGBY-JONES, P., DALTON, D. A. G., AND PAYNE, P. M.—(1952) *Brit. J. Radiol.*, Supplement 4.
World Health Organisation—(1952) 'Epidemiological and Vital Statistics Report,' 5. Geneva.


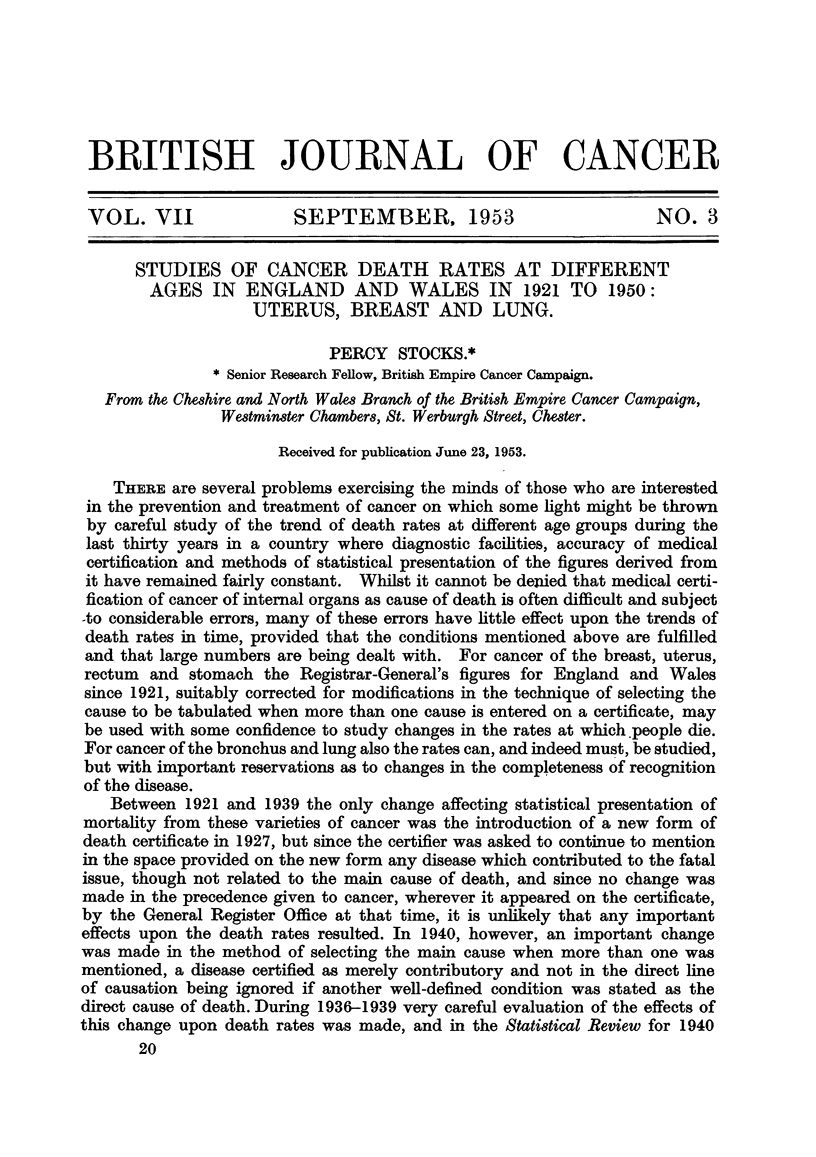

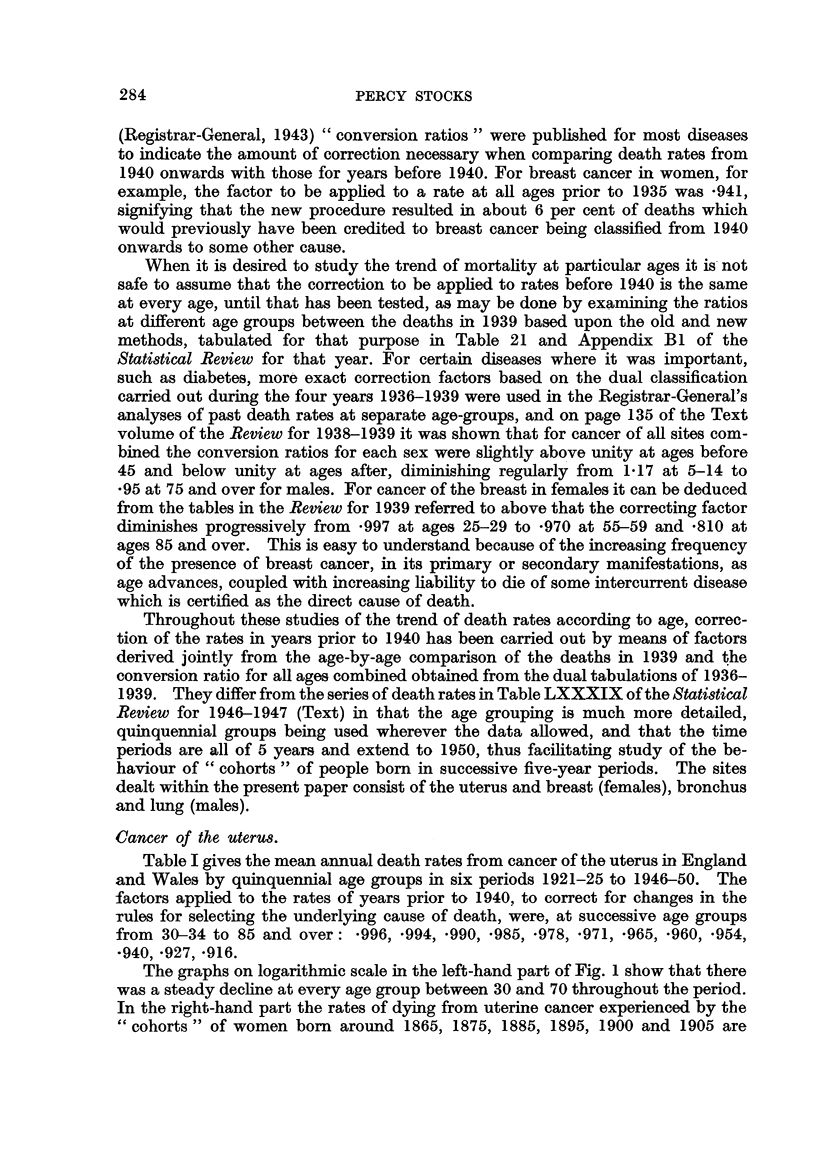

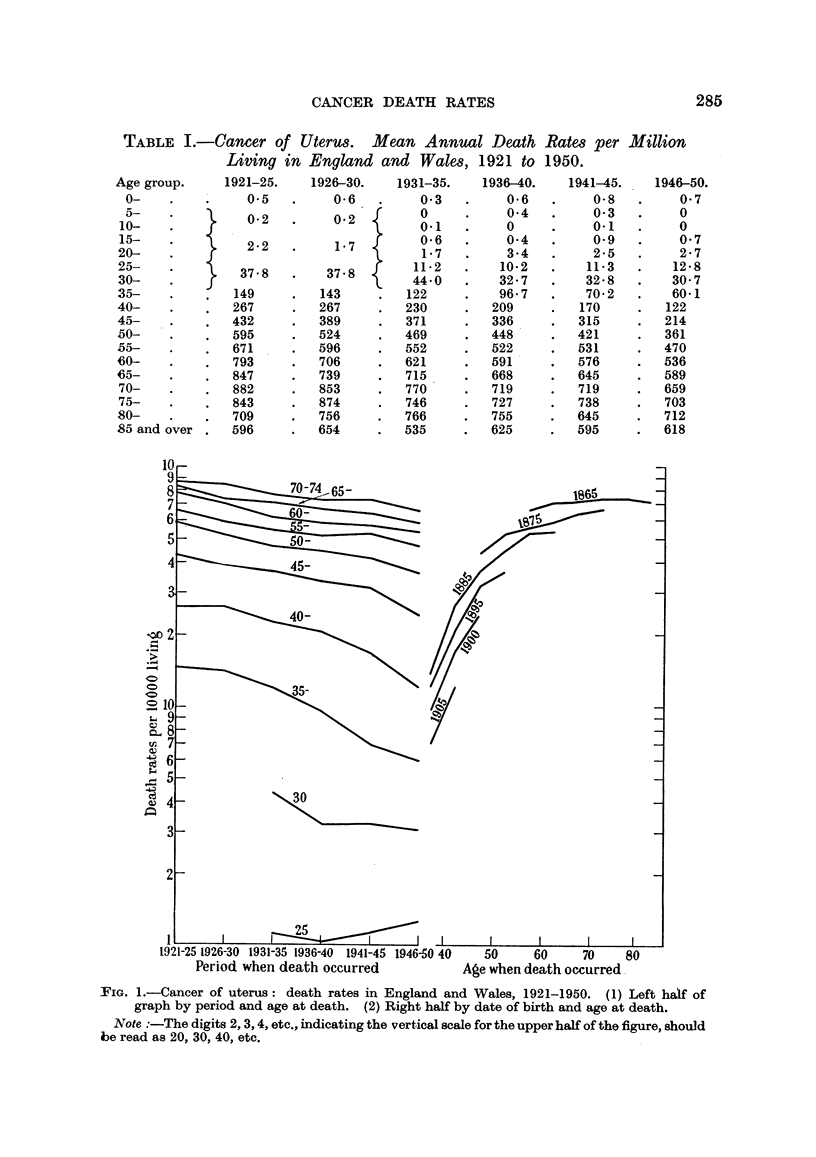

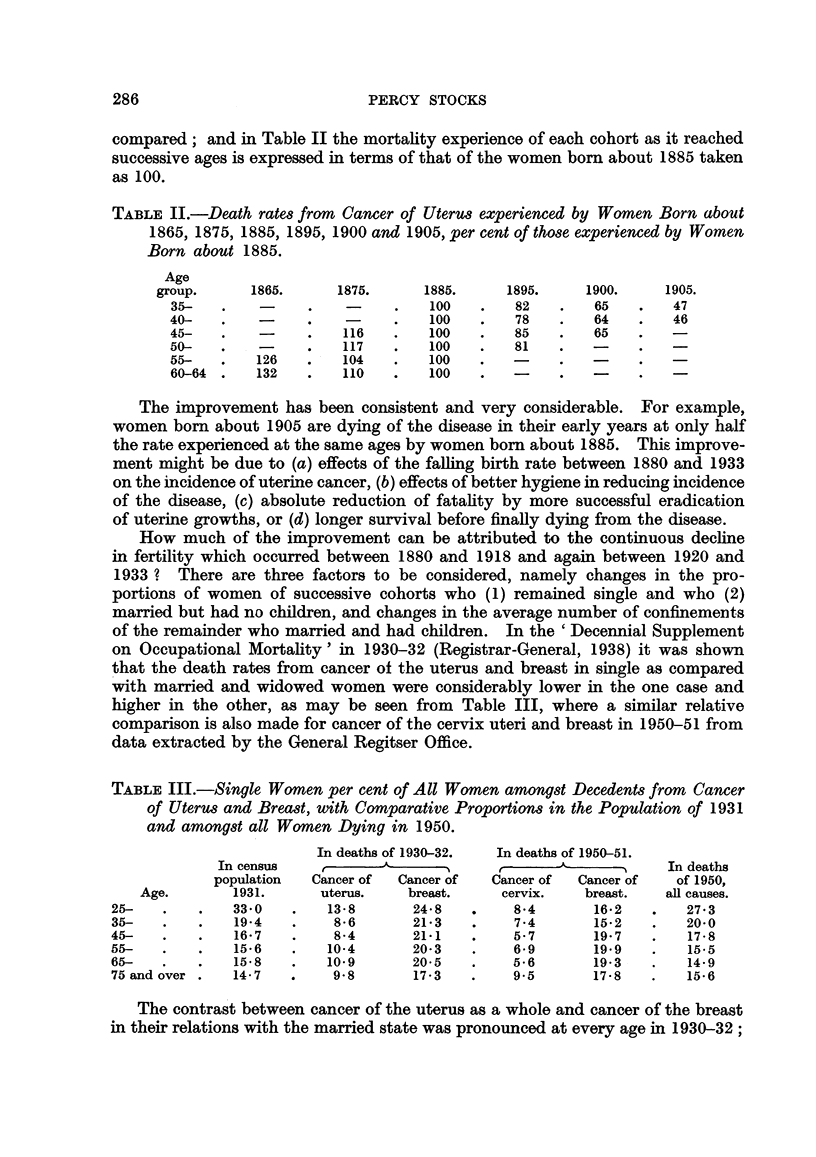

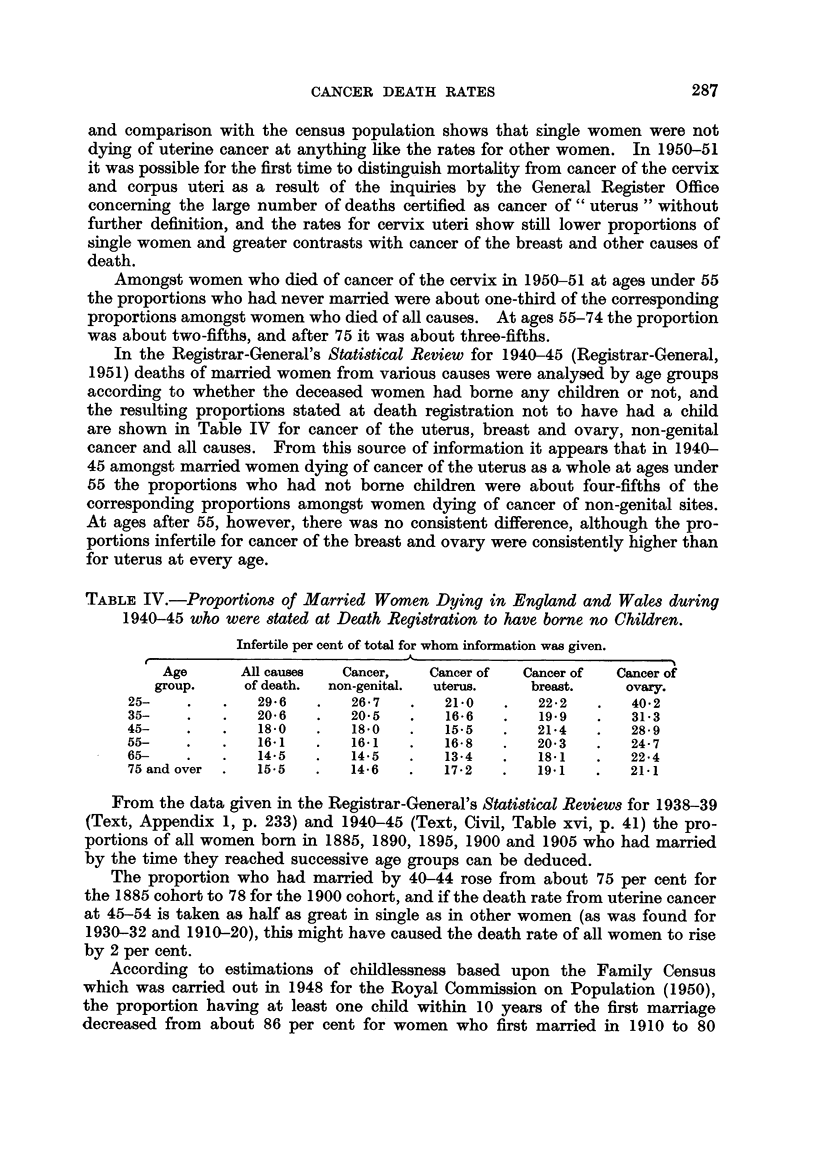

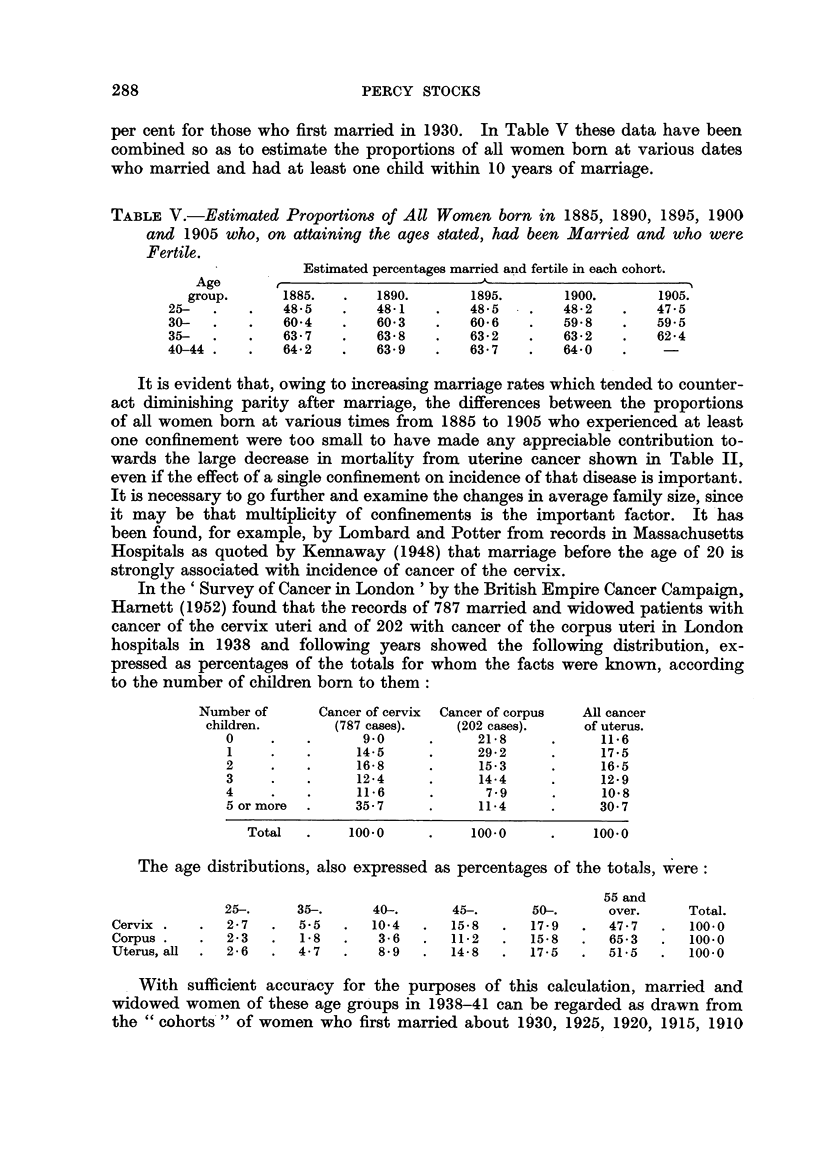

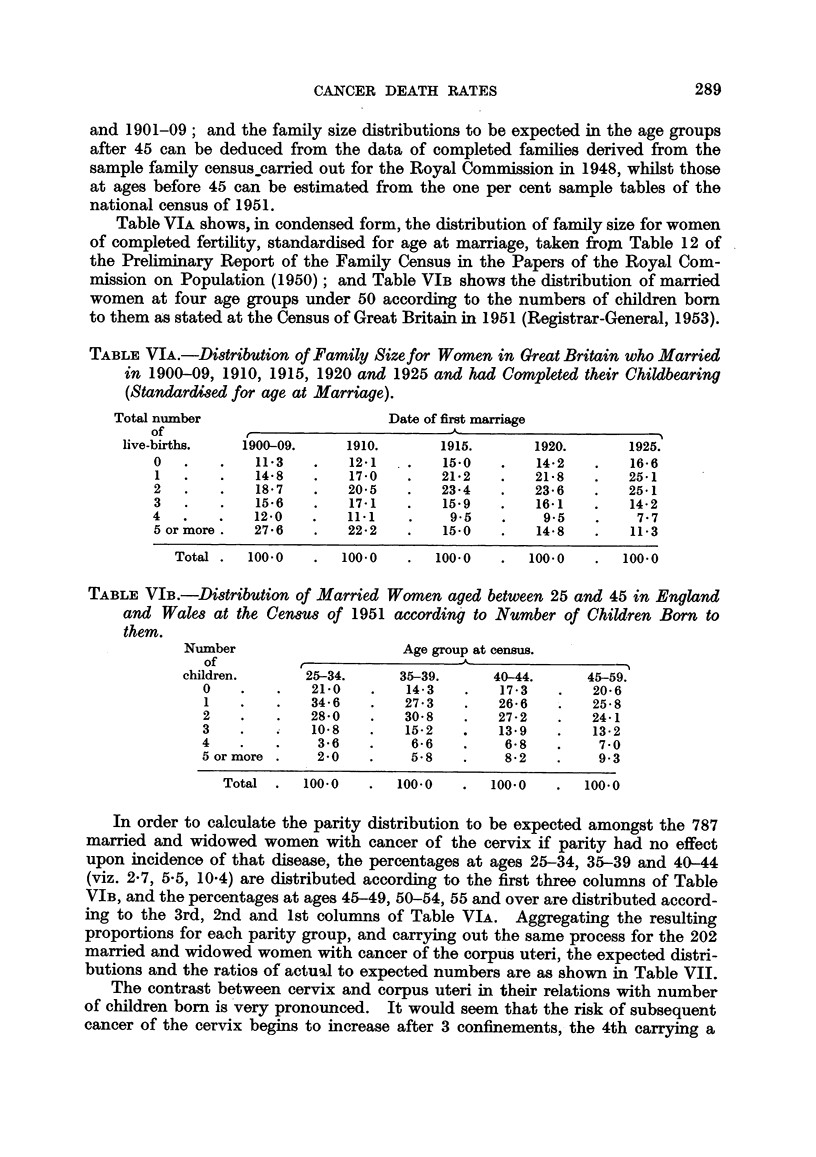

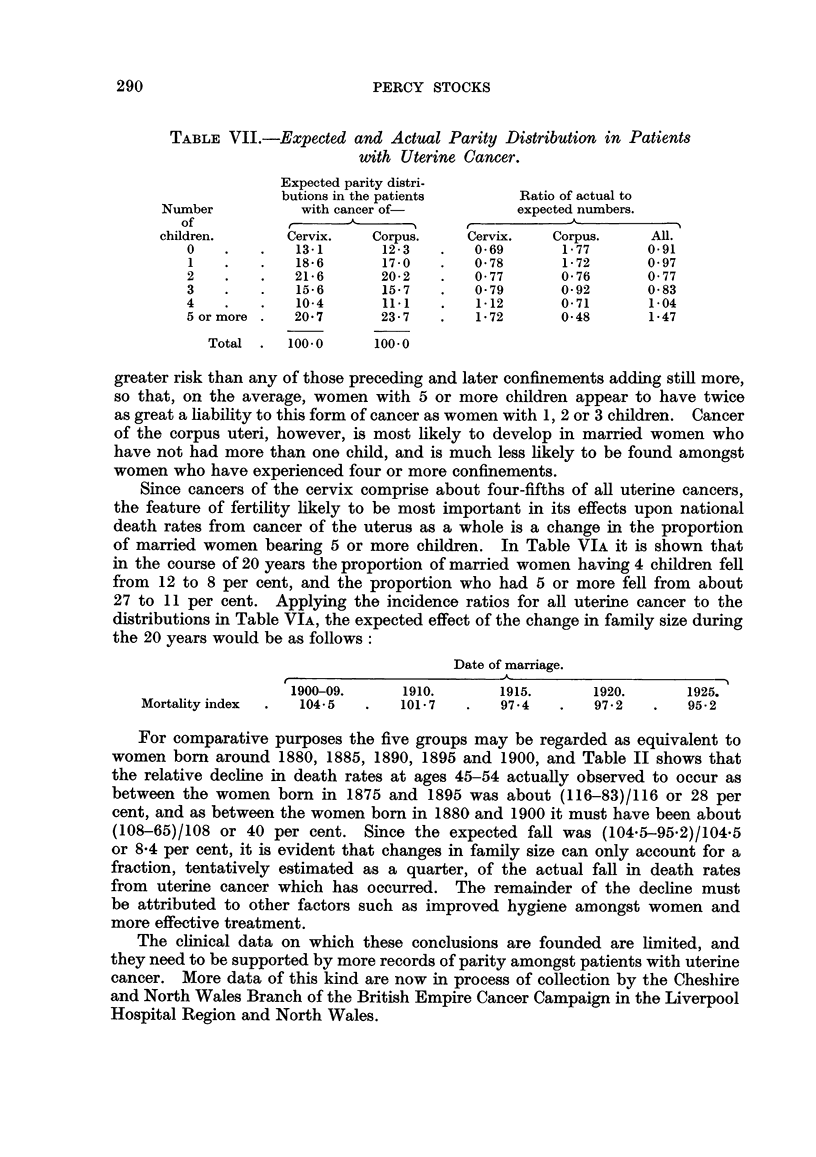

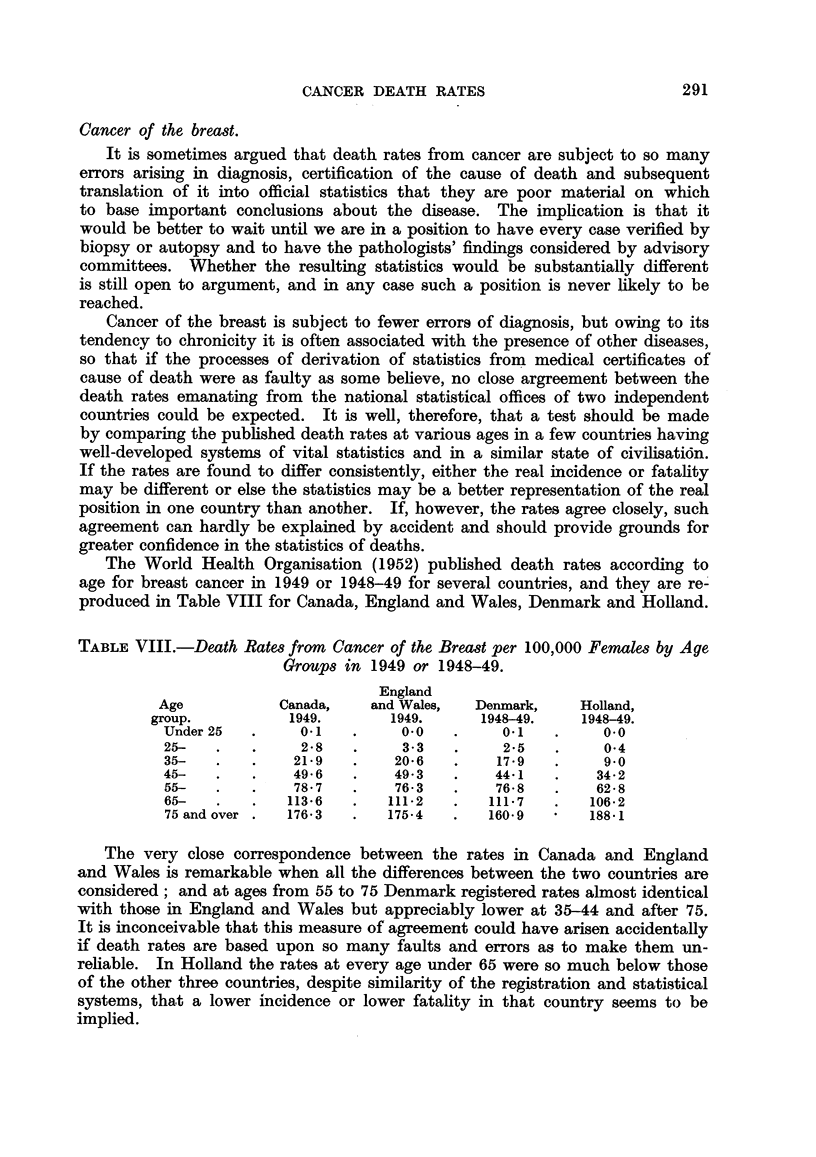

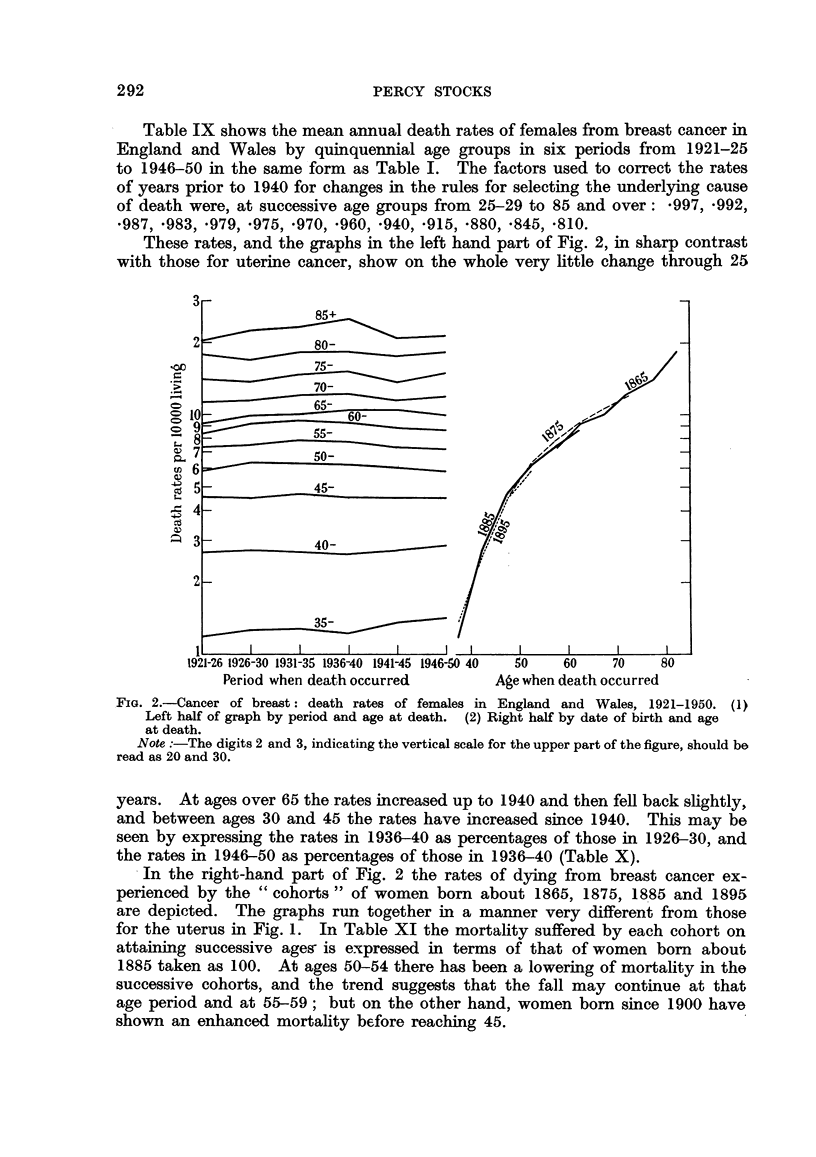

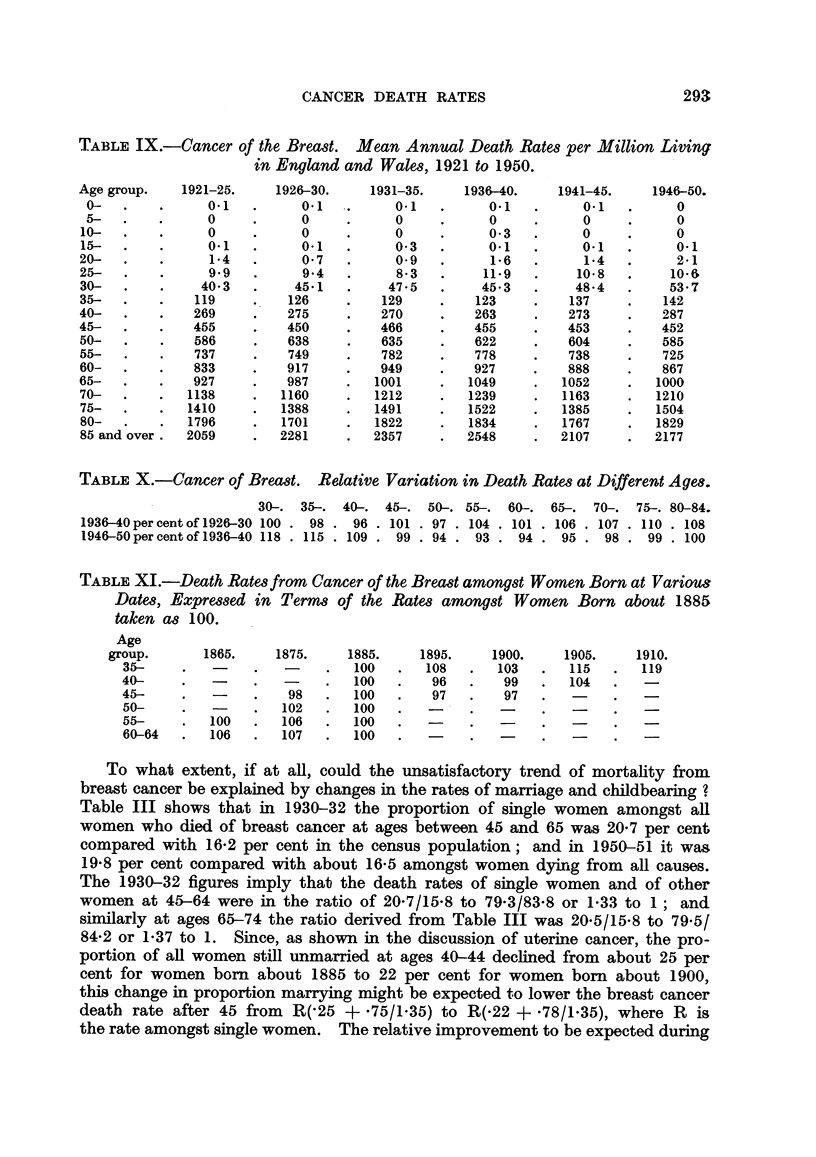

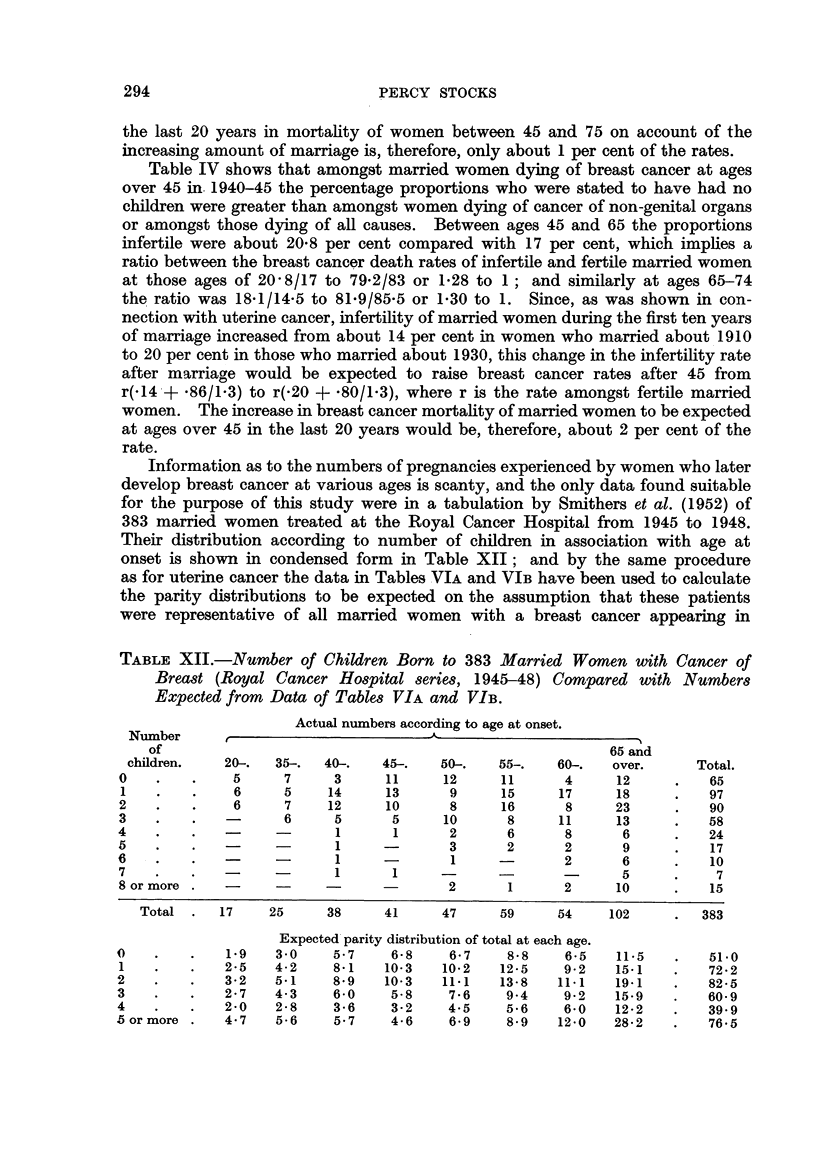

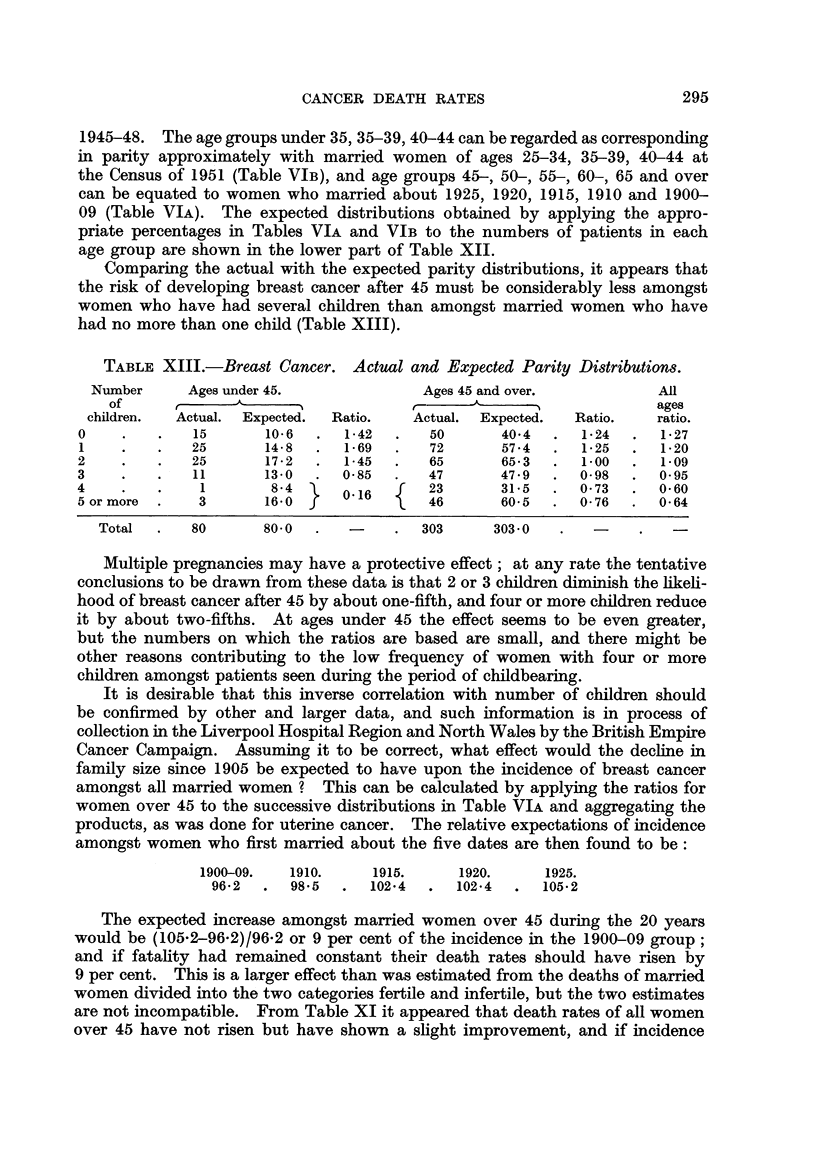

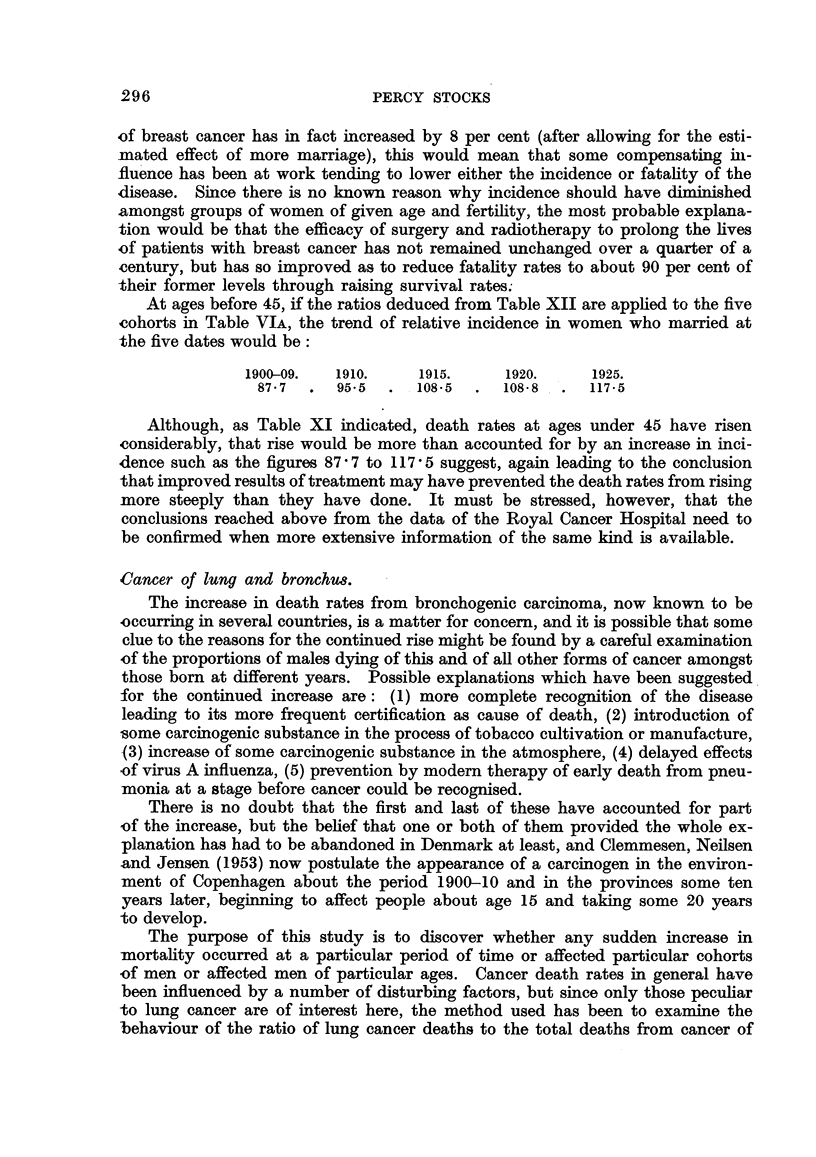

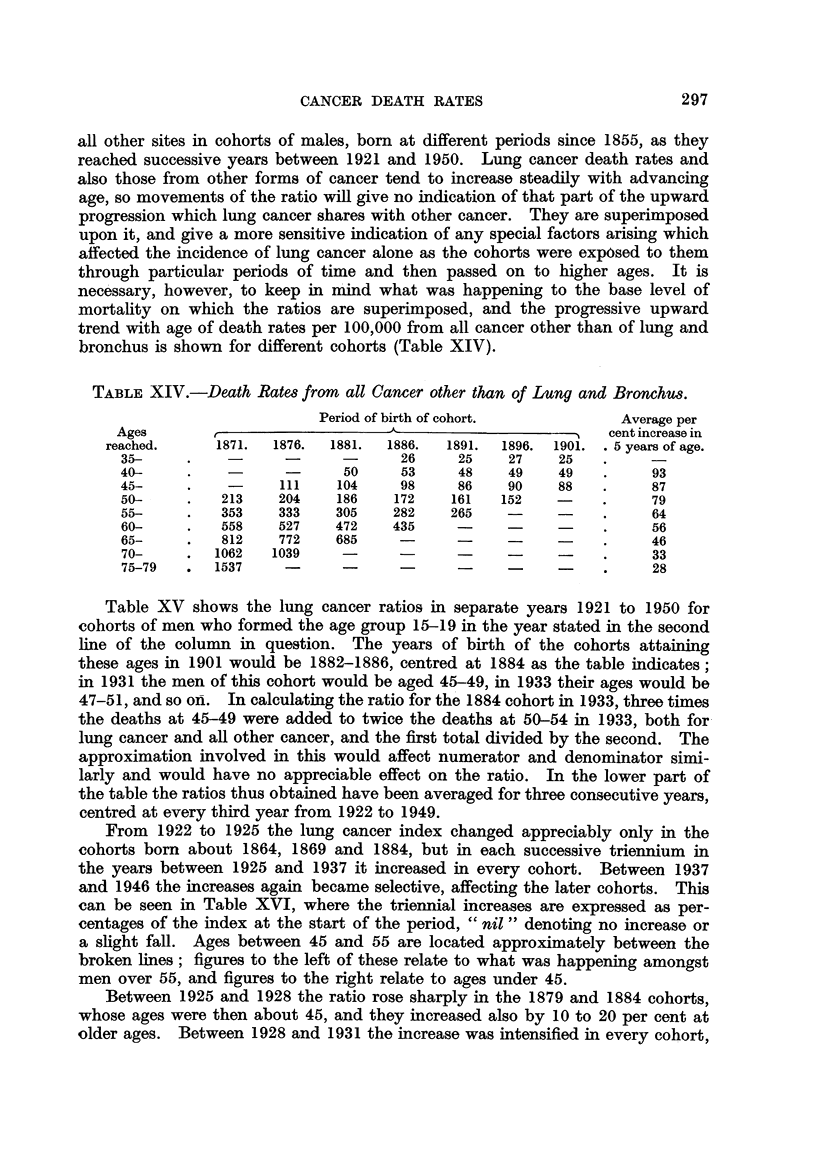

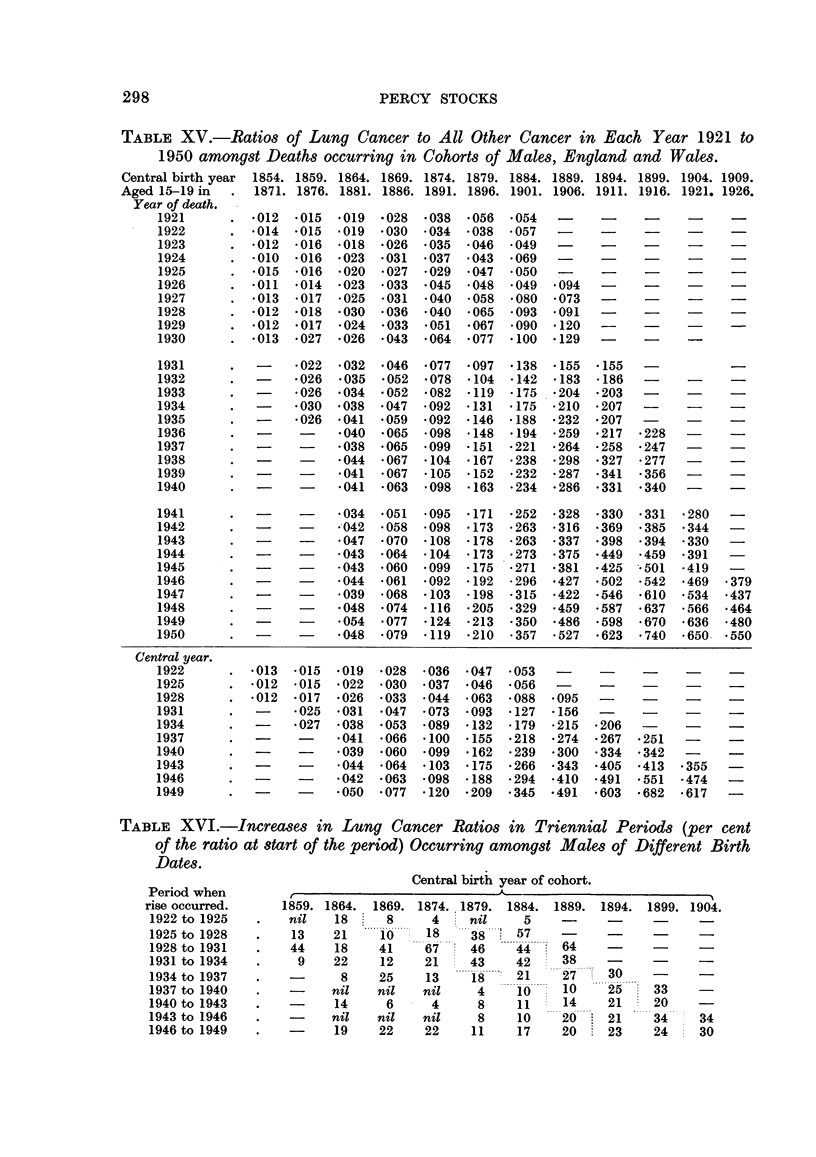

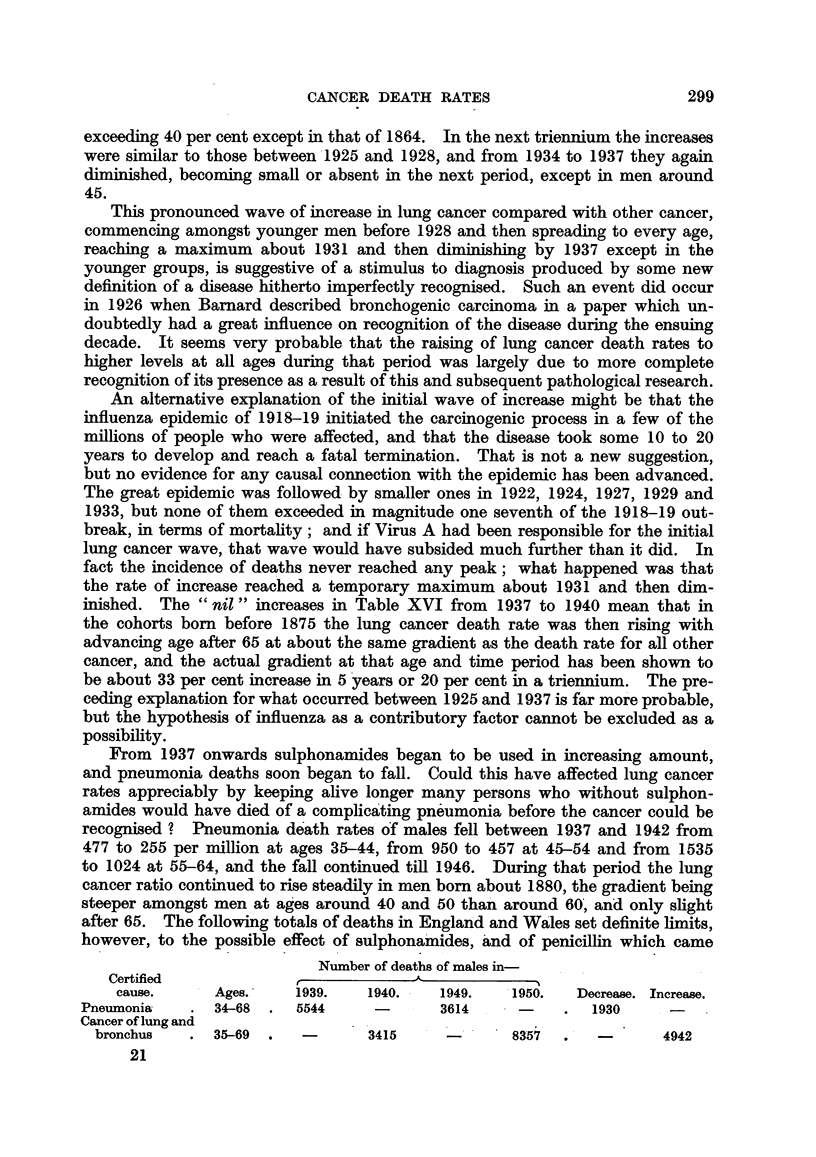

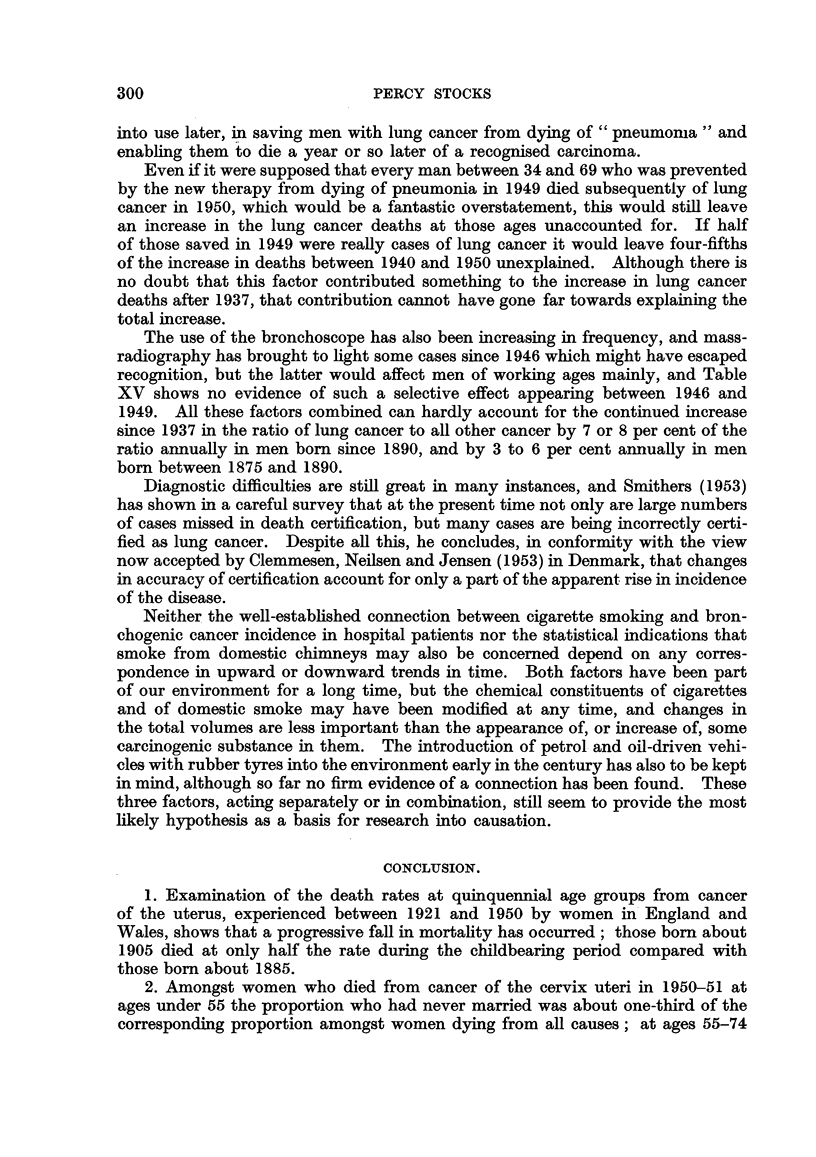

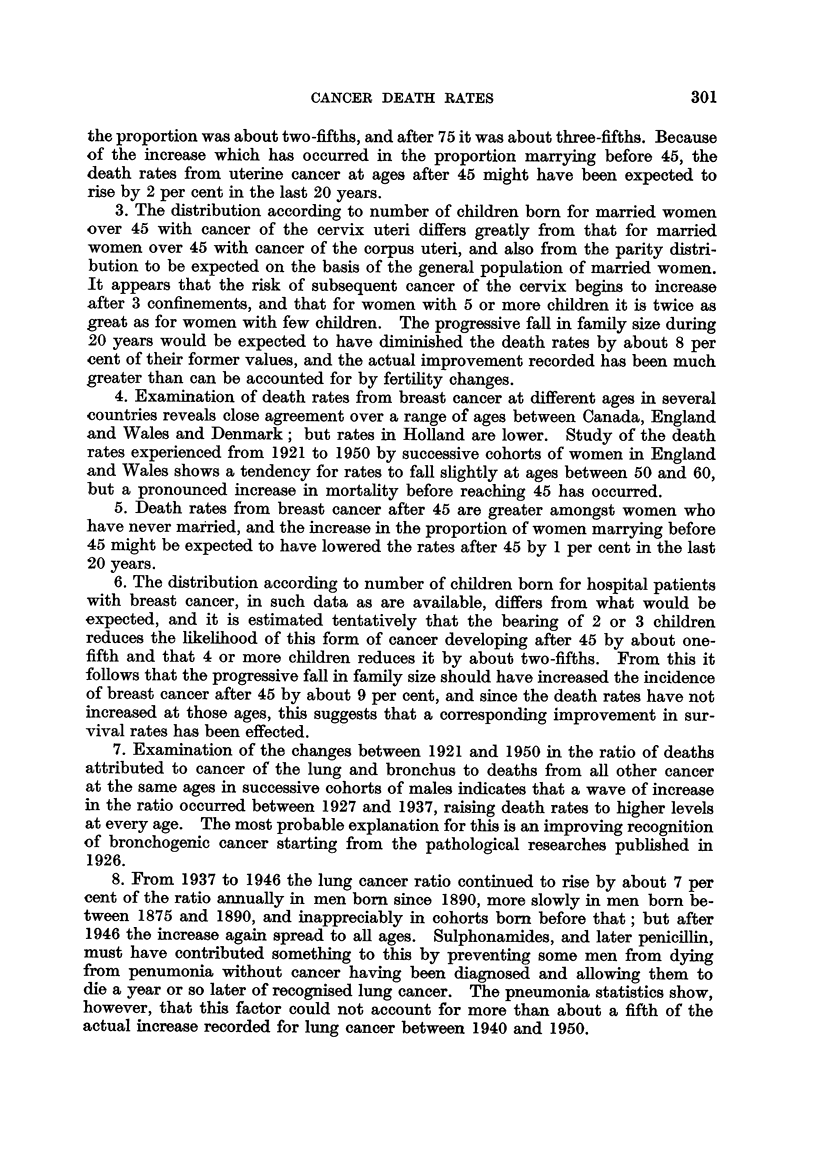

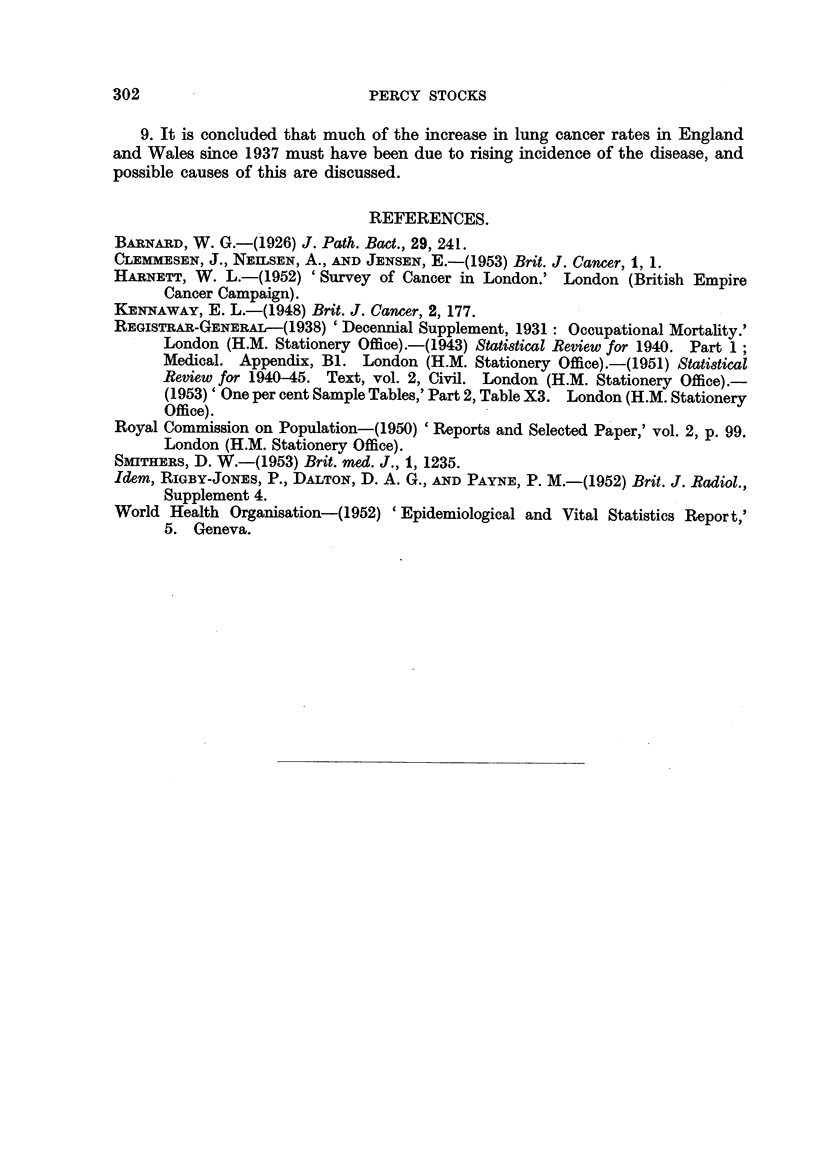


## References

[OCR_01719] SMITHERS D. W. (1953). Facts and fancies about cancer of the lung.. Br Med J.

